# A gut-derived hormone suppresses sugar appetite and regulates food choice in *Drosophila*

**DOI:** 10.1038/s42255-022-00672-z

**Published:** 2022-11-07

**Authors:** Alina Malita, Olga Kubrak, Takashi Koyama, Nadja Ahrentløv, Michael J. Texada, Stanislav Nagy, Kenneth V. Halberg, Kim Rewitz

**Affiliations:** Department of Biology, University of Copenhagen, Copenhagen, Denmark

**Keywords:** Endocrine system and metabolic diseases, Drosophila, Metabolism, Feeding behaviour, Gastrointestinal hormones

## Abstract

Animals must adapt their dietary choices to meet their nutritional needs. How these needs are detected and translated into nutrient-specific appetites that drive food-choice behaviours is poorly understood. Here we show that enteroendocrine cells of the adult female *Drosophila* midgut sense nutrients and in response release neuropeptide F (NPF), which is an ortholog of mammalian neuropeptide Y-family gut-brain hormones. Gut-derived NPF acts on glucagon-like adipokinetic hormone (AKH) signalling to induce sugar satiety and increase consumption of protein-rich food, and on adipose tissue to promote storage of ingested nutrients. Suppression of NPF-mediated gut signalling leads to overconsumption of dietary sugar while simultaneously decreasing intake of protein-rich yeast. Furthermore, gut-derived NPF has a female-specific function in promoting consumption of protein-containing food in mated females. Together, our findings suggest that gut NPF-to-AKH signalling modulates specific appetites and regulates food choice to ensure homeostatic consumption of nutrients, providing insight into the hormonal mechanisms that underlie nutrient-specific hungers.

## Abstract (web summary)

Malita, Kubrak et al. show that the gut-derived hormone neuropeptide F suppresses sugar intake and increases the consumption of protein-rich food in *Drosophila*. This gives insight into the regulation of nutrient-specific appetite that ensures appropriate food choices to meet nutritional demands.

## Main

Animals must be able to select the specific nutrients they need to consume. Food selection is governed by appetites for specific nutrients to ensure adequate ingestion of macronutrients needed to maintain nutritional homeostasis and optimal fitness^1,2^. This has given rise to the hypothesis that organisms can feel specific hungers or appetites for the type of nutrients they need^3,4^. Nutrient-specific appetite has been demonstrated in many organisms, including humans^3–6^. Such homeostatic nutrient consumption requires sensors that detect the internal nutritional state and mechanisms that translate this information into changes in feeding decisions. Food consumption is controlled by nutritional signals from the periphery, such as the adipokine leptin and a variety of gut hormones, that act together with circulating nutrients on the brain^7^. However, the hormones and mechanisms that govern nutrient-specific appetites that drive appropriate food choices to maintain or restore homeostasis are poorly defined.

The fruit fly *Drosophila*, like mammals, regulates feeding behaviours according to internal state^1,6,8,9^. The gut is one of the largest endocrine organs, releasing a number of different hormones from specialized enteroendocrine cells (EECs) in both flies and mammals^10,11^. Gut-to-brain signalling conveys important information about the nutritional nature of the intestinal contents, and enteric nutrient-sensing and signalling play key roles in regulating food intake^12,13^. For example, in the nutrient-deficient state, orexigenic or hunger signals from the mammalian gut such as the hormone ghrelin drive appetite to promote food consumption. Conversely, in response to food consumption, the mammalian gut releases glucagon-like peptide 1 (GLP-1), which acts as a satiety signal that reduces further food intake. Such satiety signals prevent excess nutrient intake, which can lead to the development of obesity and associated metabolic disorders, and GLP-1 therapy is effective in reducing body weight by lowering appetite^14^. The fly gut is structurally similar to the mammalian gastrointestinal tract, and many gut-derived hormones are evolutionarily conserved^15,16^, making *Drosophila* an attractive model for unravelling the signals by which the gut controls feeding decisions and sex differences in feeding behaviour. Indeed, a great deal has been learned about gut-derived hormonal signalling in this system^17–21^.

Although substantial progress has been made in understanding the gut-hormonal signalling that controls metabolism^18,20,21^, much less is known about how the gut communicates the presence or absence of specific nutrients to adjust food choice, and gut-derived signals that regulate appetite towards specific nutrients have not been described. Here, we show that EECs in the adult female *Drosophila* gut sense sugar and in response release neuropeptide F (NPF), an ortholog of mammalian neuropeptide Y (NPY) hormones. NPF acts via several routes of tissue crosstalk to suppress sugar appetite and promote intake of protein-rich food in mated females, suggesting that NPF is important for regulation of food choices and prevention of excessive sugar consumption, which has been linked to obesity.

## Results

### Midgut NPF suppresses sugar intake and energy breakdown

**Fig. 1:**
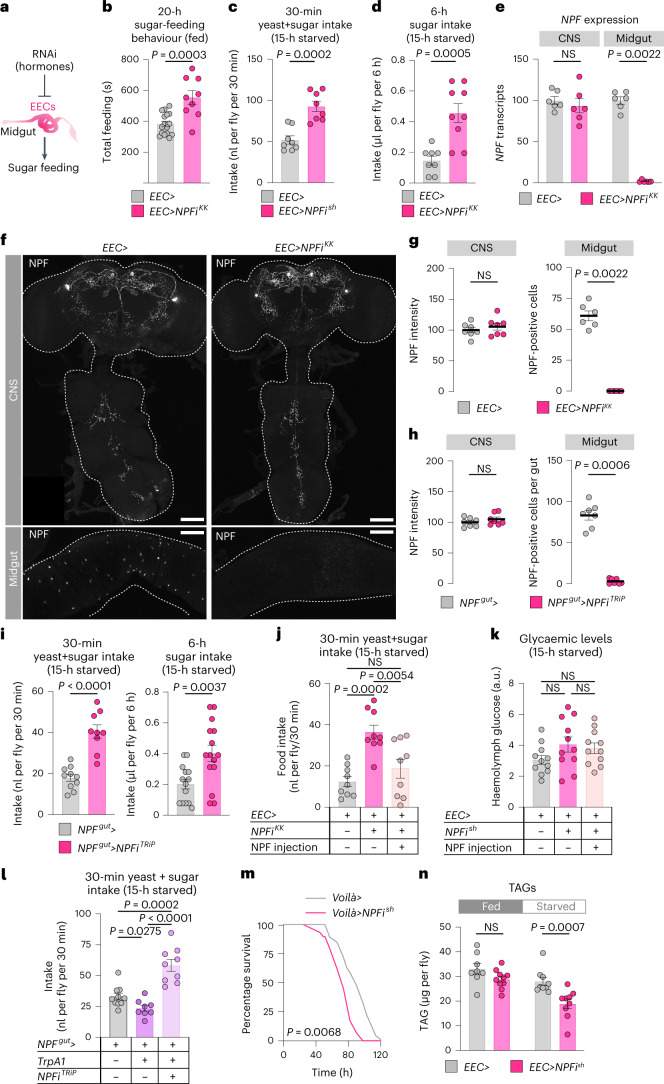
Gut-derived NPF regulates sugar intake and metabolism in mated females. **a**, Sugar feeding in mated females with RNAi-mediated knockdown of hormones and transporters in the EECs of the midgut. **b**, Total time feeding using FLIC; *n* = 16 *EEC*>, *n* = 9 *EEC* > *NPFi*^*KK*^. **c**,**d**, Consumption (**c**) of sugar+yeast food (9% sugar and 8% yeast) determined by dye assay, and of 10% sugar (**d**) measured by CAFÉ assay; **c**, *n* = 8 *EEC*> and *EEC*>*NPFi*^*sh*^; **d**, *n* = 8 *EEC*>, *n* = 9 *EEC*>*NPFi*^*KK*^. **e**, Conditional *NPF* knockdown with *EEC*> affects the EECs but not the CNS (brain and VNC); *n* = 6 biological replicates from tissues pooled from six animals for each condition. **f**,**g**, NPF immunostaining of CNS and midgut, quantified in **g**; *n* = 7 CNS, *n* = 6 midguts. Scale bars, 50 μm. **h**, Quantification of images represented in Extended Data Fig. 1i, immunostaining of *NPF* knockdown using the *NPF*> driver with pan-neuronal *GAL80* (*R57C10-GAL80; NPF*>– together, *NPF*^*gut*^>) of midgut EECs and CNS; *n* = 7 tissues each. **i**, Intake measured by dye-consumption and CAFÉ assays. Left *n* = 10 *NPF*^*gut*^>, *n* = 9 *NPF*^*gut*^>*NPFi*^*TRiP*^; right *n* = 14 *NPF*^*gut*^>, *n* = 15 *NPF*^*gut*^>*NPFi*^*TRiP*^. **j**,**k**, Consumption and glycaemic levels after injection of NPF peptide into the haemolymph. **j**, *n* = 9 each. **k**, *n* = 11 each. **l**, Thirty-minute food intake measured by dye assay during activation of NPF^+^ EECs using the heat-sensitive TrpA1 channel; *n* = 10 *NPF*^*gut*^>, *n* = 8 *NPF*^*gut*^>*TrpA1*, *n* = 9 *NPF*^*gut*^>*TrpA1, NPFi*^*TRiP*^. **m**, Survival under starvation. **n**, TAG levels; *n* = 8 fed *EEC*>, *n* = 10 fed *EEC*>*NPFi*^*KK*^, *n* = 9 starved *EEC*>, *n* = 9 starved *EEC*>*NPFi*^*KK*^. All animals were mated females. Bars represent mean ± s.e.m. NS, not significant. **b**–**d**,**i**, Two-tailed unpaired Student’s *t*-test. **e**,**g**,**h**,**n**, Two-tailed unpaired Mann–Whitney *U*-test. **j**–**l**, One-way ANOVA with Tukey’s multiple-comparisons test. **m**, Kaplan–Meier log-rank tests. Source data

**Extended Data Fig. 1:**
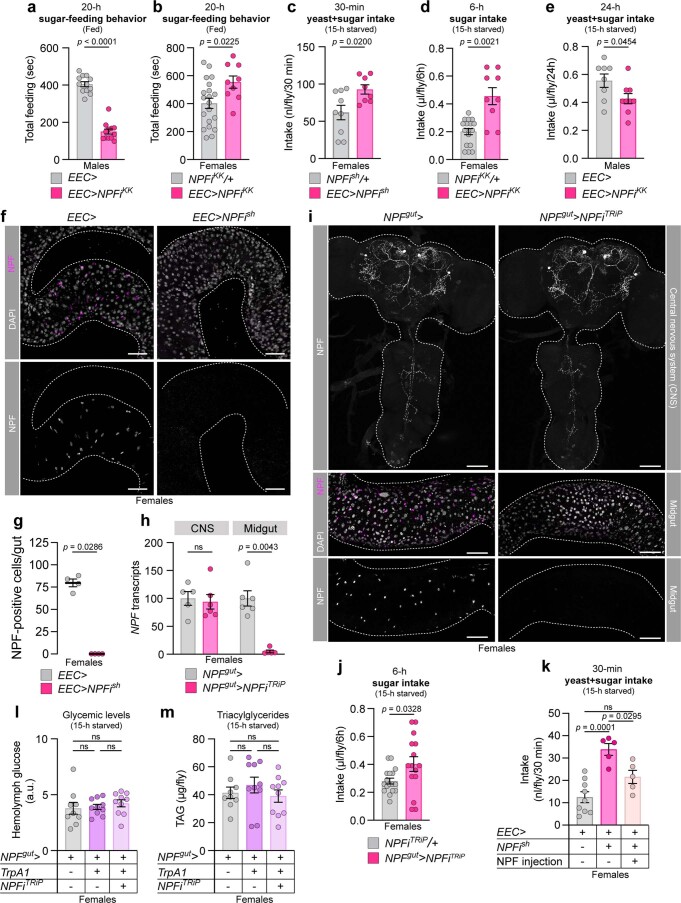
*NPF* knockdown efficiently depletes NPF specifically in the midgut EECs and affects food intake. (**a**) Total time feeding using FLIC; n = 12. (**b**) Time spent feeding determined by FLIC; n = 20 *NPFi*^*KK*^*/+*, n = 9 *EEC* > *NPFi*^*KK*^. (**c**) Amount of sugar+yeast solid food (9% sugar + 8% yeast) consumed, by dye assay; n = 9 *NPFi*^*sh*^*/+*; n = 8 *EEC* > *NPFi*^*sh*^. (**d**) Consumption of 10% sugar measured by CAFÉ assay; n = 17 *NPFi*^*KK*^*/+* and n = 9 *EEC* > *NPFi*^*KK*^. (**e**) Consumption of sugar+yeast liquid food (5% sugar + 5% yeast extract) measured by CAFÉ assay; n = 8 *EEC* > , n = 9 *EEC* > *NPFi*^*KK*^. (**f**,**g**) NPF immunostaining in the midgut of mated females, quantified in (**g**); n = 4 guts. Scale bars, 50 μm. (**h**) Knockdown using the *R57C10-GAL80, NPF* > (*NPF*^*gut*^ > ) driver with *NPFi*^*TRiP*^ affects *NPF* transcripts in mated female guts but not the CNS (brain and ventral nerve cord), n = 5 *NPF*^*gut*^ > CNS samples, n = 6 *NPF*^*gut*^ > *NPFi*^*TRiP*^ CNS samples, n = 6 *NPF*^*gut*^ > midgut samples, n = 5 *NPF*^*gut*^ > *NPFi*^*TRiP*^ midgut samples, each replicate containing tissues from 6 animals. (**i**) NPF immunostaining of CNS and midguts from mated females, quantified in Fig. 1 h. Scale bars, 50 μm. (**j**) Consumption of 10% sugar-water measured by CAFÉ assay; n = 17 *NPFi*^*TRiP*^*/+*, n = 15 *NPF*^*gut*^ > *NPFi*^*TRiP*^. (**k**) Food intake after injection of NPF peptide into the haemolymph measured by dye assay; n = 9 *EEC* > , n = 5 *EEC* > *NPFi*^*sh*^, n = 5 *EEC* > *NPFi*^*sh*^ with NPF injection. (**l**,**m**) Hemolymph glucose (**l**) and whole-body TAG (**m**) levels after 30-minute incubation at 29 °C for TrpA1 activation; n = 9 *NPF*^*gut*^ > , n = 10 *NPF*^*gut*^ > *TrpA1*, n = 10 *NPF*^*gut*^ > *TrpA1* + *NPFi*. All females were mated. Bars represent mean±SEM. ns, non-significant. **a, b, c, e, j:** Two-tailed unpaired Student’s t test. **d, g, h:** Two-tailed unpaired Mann–Whitney U test. **k:** One-way ANOVA with Tukey’s multiple-comparisons test. **l, m:** Kruskal-Wallis nonparametric ANOVA with Dunn’s multiple-comparisons test. Source data

To identify gut-derived hormones and nutrient-sensing mechanisms that regulate feeding, we performed an in vivo RNA-interference screen of secreted factors and receptors in adult *Drosophila*. We focused on the EECs, which produce a variety of factors that play key roles in the coordination of food intake and metabolism^12,18,20,21^. We examined the effect of adult-restricted, EEC-specific gene knockdown on the sugar-water feeding behaviour of fed males and females (Fig. 1a) using the fly liquid-food interaction counter (FLIC) system, which allows automated monitoring of *Drosophila* feeding behaviours^22^. We used the driver *voilà-GAL4* to target the RNAi effect to the EECs, in combination with ubiquitously expressed temperature-sensitive GAL80 (*Tub-GAL80*^*TS*^), together referred to as *EEC*> hereafter, which allowed us to induce gene silencing only in the adult stage^18,20^. Among our hits was the peptide NPF, knockdown of which increased the feeding time of mated females on sugar-only food while decreasing males’ sugar-interaction time (Fig. 1b and Extended Data Fig. 1a). To rule out contributions of the UAS transgene itself to the phenotype, we crossed the *UAS-NPF-RNAi*^*KK*^ (*NPFi*^*KK*^) line to the control *w*^*1118*^ background. This genotype showed results similar to those seen with the driver control (Extended Data Fig. 1b). This suggests that lack of NPF production in the EECs of mated females enhances their interest in or motivation to feed on sugar.

Knockdown of gut *NPF* throughout development has recently been associated with increased consumption of food containing both sugar and yeast in virgin female adults^21^. We therefore analyzed whether EEC-derived NPF also regulates intake of sugar+yeast food in mated females. We measured short-term (30-minute) food intake using a dye-consumption assay with standard adult fly food^23^ containing both sugar (9%) and yeast (8%). To measure short-term intake, we preconditioned animals by fasting them for 15 hours to increase consumption. We confirmed that, like virgins, mated females with adult-restricted EEC knockdown of *NPF* also consumed significantly more sugar+yeast food than controls (Fig. 1c and Extended Data Fig. 1c). We also applied the capillary feeder (CAFÉ) assay^24^ to quantify sugar intake over a longer period. Before this assay as well, we exposed animals to a 15-hour period of fasting to enhance consumption. Mated females with adult-restricted EEC knockdown of *NPF* consumed significantly more sugar over the 6-hour period than controls (Fig. 1d and Extended Data Fig. 1d). Since the sugar-feeding phenotype was observed in animals whether they were fully fed or preconditioned by 15-hour fasting (Fig. 1b,c,d), we chose to use animals that were 15-hour fasted for consistency in the following feeding assays, since this allows robust measurements in short-term food intake assays as well as longer-term feeding assays. Conversely, in males, loss of *NPF* in the EECs led to reduced food consumption over an even longer period of 24 hours (Extended Data Fig. 1e). Together, these results indicate that gut-derived NPF suppresses sugar intake in females.

To attribute these effects specifically to EEC-derived NPF, we measured the expression of *NPF* in dissected midguts and central nervous systems (CNS; brain and ventral nerve cord, VNC). *NPF* transcript levels were strongly reduced in the female midgut when *EEC*> was used to drive knockdown of *NPF*, whereas expression in the CNS was unaltered, which was confirmed by immunostainings (Fig. 1e,f,g and Extended Data Fig. 1f,g). To further support this, we used a second driver, *NPF::2A::GAL4* (*NPF*>), a CRISPR-mediated insertion of *T2A::GAL4* into the native *NPF* locus that drives GAL4 expression in only NPF-producing cells^25^. Since NPF is expressed in both neurons and EECs, we used pan-neuronal *R57C10-GAL80*, an optimized *nSyb-GAL80* variant that suppresses neuronal GAL4 (ref. ^18^), to suppress the GAL4 activity of *NPF*> in the nervous system. We confirmed that this driver combination (*R57C10-GAL80; NPF*>), referred to hereafter as *NPF*^*gut*^>, efficiently knocks *NPF* down in the midgut without affecting CNS expression (Fig. 1h and Extended Data Fig. 1h,i). Knockdown of *NPF* using this gut NPF-specific driver caused a marked increase in intake of sugar+yeast food measured over 30 minutes and in consumption of sugar-only medium measured over 6 hours after 15-hour starvation (Fig. 1i and Extended Data Fig. 1j). Taken together, these data indicate that EEC-specific loss of *NPF* is responsible for the observed feeding phenotypes and indicate that gut NPF acts as a satiety signal that inhibits sugar consumption.

To examine the ability of NPF to promote satiety, we injected synthetic NPF peptide into circulation in mated females. EEC-specific knockdown of *NPF* induced hyperphagia, which was blocked by NPF injection (Fig. 1j and Extended Data Fig. 1k). NPF injection did not affect haemolymph sugar levels (Fig. 1k), indicating that the observed feeding effect was not a consequence of alterations in glycaemic levels. Next, we expressed the thermosensitive Transient receptor potential A1 (TrpA1) cation channel^26^ in the NPF^+^ EECs to enable induction of NPF release. Incubation at 29 °C, which induces TrpA1-mediated peptide release, inhibited food intake, an effect that was abolished by simultaneous *NPF* knockdown (Fig. 1l). These NPF-induced changes in food intake were likewise not associated with altered triacylglyceride (TAG) or circulating sugar levels (Extended Data Fig. 1l,m), supporting a direct role for gut-derived NPF in governing feeding behaviour, rather than effects of NPF on metabolism that then lead secondarily to altered behaviour. Together, these results indicate that NPF from these EECs is both necessary and sufficient to inhibit food intake and prevent food overconsumption.

**Extended Data Fig. 2:**
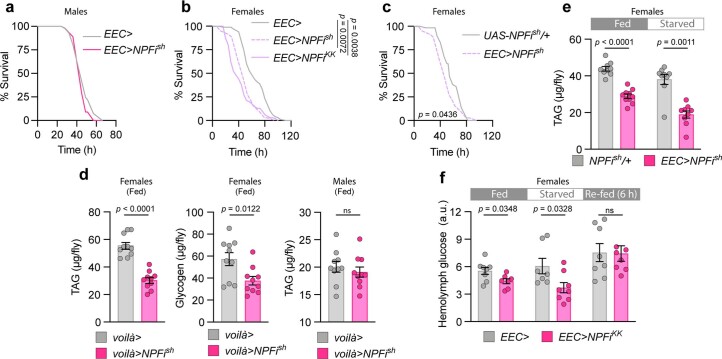
Enteroendocrine NPF regulates systemic metabolism and resistance to nutritional stress. (**a**-**c**) Survival during starvation; in (**a**), n = 41 *EEC* > males, n = 45 *EEC* > *NPFi*^*sh*^ males; in (**b**), n = 130 *EEC* > , n = 122 *EEC* > *NPFi*^*sh*^, n = 94 *EEC* > *NPFi*^*KK*^; in (**c**), n = 50 *UAS-NPFi*^*sh*^*/+*, n = 122 *EEC* > *NPFi*^*sh*^. (**d**) TAG and glycogen levels in animals with gut *NPF* knockdown during development; n = 10. (**e**) TAG levels in the fed condition and following 24-hour starvation; n = 9 fed *NPFi*^*KK*^*/+*, n = 10 fed *EEC* > *NPFi*^*KK*^, n = 9 starved *NPFi*^*KK*^*/+*, n = 9 starved *EEC* > *NPFi*^*KK*^. (**f**) Hemolymph sugar levels in the fed condition, after 24 hours’ starvation, and after 6 hours of re-feeding from the 24-hour-starved condition; n = 8 fed *EEC* > , n = 7 fed *EEC* > *NPFi*^*KK*^, n = 7 starved *EEC* > , n = 8 starved *EEC* > *NPFi*^*KK*^, n = 8 re-fed *EEC* > , n = 7 re-fed *EEC* > *NPFi*^*KK*^. All females were mated. Bars represent mean±SEM. ns, non-significant. **a, b, c:** compared using Gehan-Breslow-Wilcoxon test**. d, e (left), f:** Two-tailed unpaired Student’s t test. **e (right):** Two-tailed unpaired Mann-Whitney U test. Source data

Feeding behaviours are tightly coordinated with physiology to maintain metabolic balance. Our findings indicate that NPF acts as a satiety signal, which suggests that it should act after a meal. In this scenario NPF would be expected to promote storage and inhibit mobilization of energy. As an indirect measure of energy storage and mobilization, we first assessed animals’ starvation resistance. *NPF* knockdown in the EECs throughout development (*voilà>* without *Tub-GAL80*^*TS*^ (Fig. 1m)), as well as adult-restricted RNAi (Extended Data Fig. 2a,b,c), led to a decrease in starvation resistance in females but not males, in line with a recent study linking gut NPF to metabolic programs associated with energy storage^21^. Consistent with their shortened starvation survival, we found that females with constitutive (Extended Data Fig. 2d) or adult-restricted (Fig. 1n and Extended Data Fig. 2e) EEC knockdown of *NPF* showed a decrease in both TAG and glycogen levels, whereas TAG levels were not affected by EEC-specific *NPF* loss in males. These observations suggest that although NPF does affect metabolism in the adult stage, it also regulates early life history in ways that affect the adult. We found that haemolymph glucose levels increased more after re-feeding in animals with EEC suppression of *NPF*, consistent with their increased sugar consumption (Extended Data Fig. 2f), and showing that the mechanisms of sugar absorption and transport into circulation are functional. Together our findings indicate that, in addition to the metabolic findings described recently^21^, a main function of EEC-derived NPF in the adult stage is the regulation of feeding, particularly the inhibition of sugar intake in mated females.

### Gut NPF suppresses sugar intake and regulates food choice

**Fig. 2:**
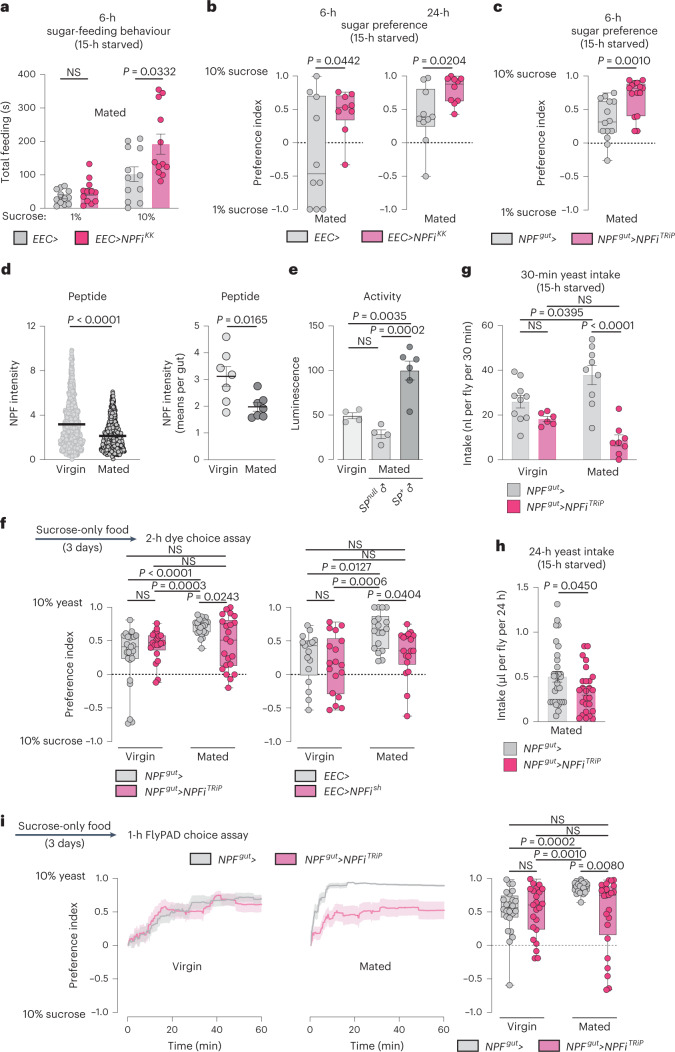
Mating induces gut NPF that suppresses sugar appetite and promotes intake of protein-rich yeast food in females. **a**, Time spent feeding on 1 or 10% sucrose using FLIC; all *n* = 12 animals. **b**,**c**, Preference between 1 versus 10% sugar measured over 6 or 24 hours by CAFÉ assay. **b**, 6-hour preference: *n* = 10 *EEC*>, *n* = 9 *EEC*>*NPFi*^*KK*^. 24-hour preference: *n* = 10 each. **c**, *n* = 14 *NPF*^*gut*^>, *n* = 15 *NPF*^*gut*^>*NPFi*^*TRiP*^. **d**, Midgut NPF staining intensity in fed females on a per-cell basis and on a per-gut basis; *n* = 703 cells from seven guts for virgins, *n* = 883 cells from seven guts from mated females. **e**, NPF-cell activity measured in dissected midguts (two midguts per replicate) using a luciferase-based CaLexA calcium-reporter system (*NPF*>*LexA::NFAT::VP16; LexAop-luciferase*); *n* = 4 from fed virgins, *n* = 4 from fed females mated to SP-deficient males (*SP*^*0*^/*Df(3L)delta130*), *n* = 6 from fed females mated to SP^+^ males. **f**, Consumption preference for 10% sucrose versus 10% yeast after 3 days of yeast deprivation (3 days on sucrose-only medium) using two-choice dye assay; *n* = 25 *NPF*^*gut*^> virgins, *n* = 23 *NPF*^*gut*^>*NPFi*^*TRiP*^ virgins, *n* = 24 *NPF*^*gut*^> mated females, *n* = 22 *NPF*^*gut*^>*NPFi*^*TRiP*^ mated females, *n* = 17 *EEC*> virgins, *n* = 18 *EEC*>*NPFi*^*sh*^ virgins, *n* = 18 *EEC*> mated females, *n* = 18 *EEC*>*NPFi*^*sh*^ mated females. **g**, Yeast consumption determined by dye assay; virgins *n* = 10 *NPF*^*gut*^> virgins, n = 6 *NPF*^*gut*^>*NPFi*^*TRiP*^ virgins, *n* = 9 *NPF*^*gut*^> mated females, *n* = 8 *NPF*^*gut*^>*NPFi*^*TRiP*^ mated females. **h**, Yeast intake determined by CAFÉ; *n* = 32 *NPF*^*gut*^>, *n* = 26 *NPF*^*gut*^>*NPFi*^*TRiP*^. **i**, Cumulative behavioural preference of females for sucrose versus yeast monitored using flyPAD. Lines represent means, and shading indicates s.e.m.; *n* = 24 *NPF*^*gut*^> virgins, *n* = 23 *NPF*^*gut*^>*NPFi*^*TRiP*^ virgins, *n* = 24 *NPF*^*gut*^> mated females, *n* = 24 *NPF*^*gut*^>*NPFi*^*TRiP*^ mated females. Bars represent mean ± s.e.m. Box plots indicate minimum, 25th percentile, median, 75th percentile and maximum values. NS, not significant. **a**,**c**,**d** (left), Two-tailed unpaired Mann–Whitney *U*-test. **b**,**d** (right), **h**, Two-tailed unpaired Student’s *t*-test. **e**,**f** (right), **g**, One-way ANOVA with Tukey’s post hoc test. **f** (left), **i**, Kruskal–Wallis nonparametric ANOVA with Dunn’s multiple-comparisons test. Source data

**Extended Data Fig. 3:**
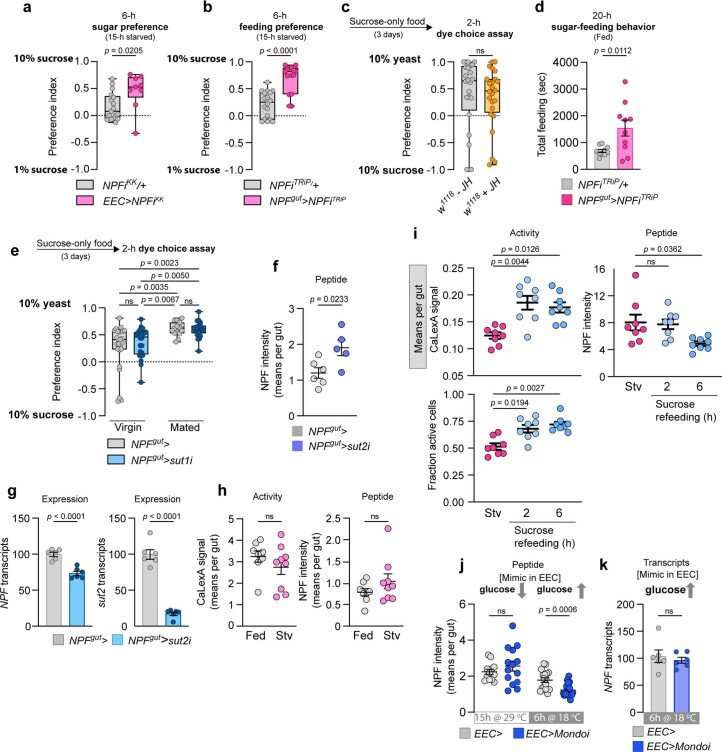
EEC-specific loss of *NPF* affects feeding and is influenced by sugar sensing. (**a**,**b**) Consumption preference for 1% *vs*. 10% sugar-water, measured by CAFÉ assay; n = 17 *NPFi*^*KK*^*/+* animals, n = 9 *EEC* > *NPFi*^*KK*^, n = 18 *NPFi*^*TRiP*^*/+*, n = 15 *NPF*^*gut*^ > *NPFi*^*TRiP*^. (**c**) Consumption preference of virgin female flies with or without JH treatment (methoprene); n = 24 each. (**d**) Feeding time on 10% sucrose measured by FLIC; n = 10 *NPFi*^*TRiP*^*/+* animals, n = 10 *NPF*^*gut*^ > *NPFi*^*TRiP*^. (**e**) Preference of virgin and mated females, measured by two-choice dye consumption assay; n = 25 *NPF*^*gut*^ > virgins, n = 24 *NPF*^*gut*^ > *sut1i* virgins, n = 15 *NPF*^*gut*^ > mated females, n = 22 *NPF*^*gut*^ > *sut1i* mated females. (**f**) Midgut NPF staining intensity with *sut2* knockdown in NPF^+^ EECs on a per-gut basis; n = 6 guts for *NPF*^*gut*^ > , n = 5 guts for *NPF*^*gut*^ > *sut2i*. (**g**) Transcript levels of *NPF* (left) and *sut2* (right) in midguts from fed mated females; n = 6 replicates containing five tissues each. (**h**) NPF intensity and NPF-cell activity (CaLexA) in fed and 24-h-starved animals on a per-gut basis; n = 8 for fed, n = 9 for 24-hours starved. (**i**) NPF intensity, NPF-cell activity (CaLexA), and fraction of CaLexA-active cells in 24-h-starved and 2-h- and 6-h-sugar-re-fed mated females measured by calcium-LexA reporter system, aggregated on a per-gut basis; n = 8 guts per condition. (**j**) NPF staining intensity in midguts of mated females with 15 hours’ EEC knockdown of *Mondo* (29 ^o^C inactivation of GAL80^TS^) and following 6-hour re-activation of Mondo expression (18 ^o^C to renature GAL80^TS^) on a per-gut basis; 15 h at 29 ^o^C: n = 12 *EEC* > , n = 14 *EEC* > *Mondoi*; 6 h at 18 ^o^C: n = 17 *EEC* > , n = 18 *EEC* > *Mondoi*. (**k**) Transcript levels of *NPF* in midguts from fed mated females; n = 6 samples of five tissues each. All animals were mated females, except in (e). Bars represent mean±SEM. Box plots indicate minimum, 25^th^ percentile, median, 75^th^ percentile, and maximum values. ns, non-significant. **a, b, c, h, j, k:** Two-tailed unpaired Mann-Whitney U test. **d, f, g:** Two-tailed unpaired Student’s t test. **e, i:** One-way Kruskal-Wallis ANOVA with Dunn’s multiple-comparisons test. Source data

Our findings suggest that NPF acts as a sugar-satiety signal. To test this hypothesis directly, we examined whether gut NPF affected animals’ preference for dietary sugar when they were given the choice between two different sucrose concentrations (1 and 10%). *NPF* knockdown in the EECs increased feeding and preference for 10% sugar in mated females (Fig. 2a,b,c and Extended Data Fig. 3a,b). These results indicate that NPF is part of a postingestion sugar-sensing mechanism required to decrease sugar appetite.

**Fig. 3:**
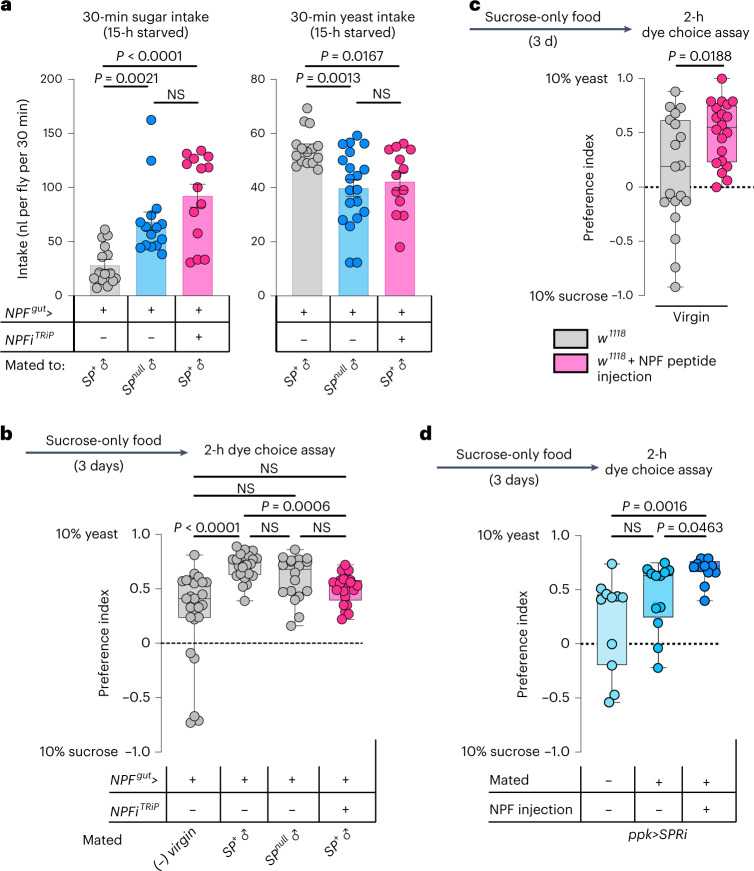
NPF regulates food choice downstream of SP signalling in mated females, and exogenous NPF promotes yeast preferences in virgins. **a**, Intake of sucrose or yeast measured by dye assay in females mated to SP-producing or SP-deficient males (*SP*^*0*^/*Df(3L)delta130*). Sucrose, *n* = 15 *NPF*^*gut*^> females mated to *SP*^*+*^ males, *n* = 15 *NPF*^*gut*^> females mated to *SP*-mutant (*SP*^*null*^) males, *n* = 14 *NPF*^*gut*^>*NPFi*^*TRiP*^ females mated to *SP*^*+*^ males. Yeast, *n* = 15 *NPF*^*gut*^> females mated to *SP*^*+*^ males, *n* = 20 *NPF*^*gut*^> females mated to *SP*-mutant males, *n* = 13 *NPF*^*gut*^>*NPFi*^*TRiP*^ females mated to *SP*^*+*^ males. **b**, Consumption preference using two-choice dye assay; *n* = 25 *NPF*^*gut*^> virgins, *n* = 24 *NPF*^*gut*^> females mated to *SP*^*+*^ males, *n* = 18 *NPF*^*gut*^> females mated to *SP*-null males, *n* = 21 *NPF*^*gut*^>*NPFi*^*TRiP*^ females mated to *SP*^*+*^ males. **c**,**d**, Consumption preference of *w*^*1118*^ virgin females with or without NPF injection (**c**) and virgin and mated females (**d**) (with and without NPF injections) with knockdown of *SPR* in Ppk^+^ neurons (*ppk*>*SPRi*) using two-choice dye assay. **c**, *n* = 19 *w*^*1118*^ virgins, *n* = 20 *w*^*1118*^ virgins with NPF injection. **d**, *n* = 11 virgin *ppk*>*SPRi* females, *n* = 12 mated *ppk*>*SPRi* females, *n* = 12 mated *ppk*>*SPRi* females with NPF injection. Bars represent mean ± s.e.m. Box plots indicate minimum, 25th percentile, median, 75th percentile and maximum values. NS, not significant. **a** (left), **b**,**d**, One-way Kruskal–Wallis ANOVA with Dunn’s multiple-comparisons test. **a**, Right, one-way ANOVA with Dunnett’s multiple-comparisons test. **c**, Two-tailed unpaired Mann–Whitney *U*-test. Source data

Given that females increase their preference for protein-rich food after mating to meet the metabolic requirements of egg production^6^, we speculated that gut NPF might be important to reduce sugar appetite and increase intake of protein-rich food in mated females. Postmating sex peptide (SP) signalling within the female induces an increased preference for yeast^6^, and this peptide has also been shown to potentiate NPF release from the midgut^17^. We confirmed that mating induces NPF secretion from the midgut by measuring EEC NPF protein levels. After mating, NPF peptide levels were reduced in the midgut, consistent with increased release (Fig. 2d). Using a luciferase-based CaLexA reporter^27^, in which calcium induces the expression of luciferase, we found that NPF^+^ EECs showed increased calcium-reporter activity after mating in an SP-dependent manner (Fig. 2e). We therefore proposed that gut-derived NPF might be involved in mediating the SP-induced increase in protein consumption in mated females. To test this possibility, we investigated whether NPF affects yeast preference by using a two-choice dye-based consumption assay to measure preference between sugar or protein-rich yeast food^6^. Animals were deprived of protein for 3 days before the experiment by keeping them on sucrose-only food to increase their preference for yeast food^6^, making any reduction in this preference easier to observe. We observed that mating increased control females’ preference for yeast food after this treatment (Fig. 2f), consistent with previous findings^6^. However, animals with EEC-specific *NPF* loss displayed a reduced preference for yeast food that did not significantly increase after mating. This indicates that EEC-derived NPF is required to inhibit sugar intake in mated females, thereby promoting consumption of protein-rich food. Consistent with this, control animals increased their yeast consumption after mating, whereas mating did not significantly increase yeast consumption in females lacking gut *NPF* that consumed less yeast (Fig. 2g,h). To further test our conjecture that gut NPF is involved in mating-induced yeast consumption, we used a second automated behaviour-monitoring apparatus, the flyPAD^28^, to measure feeding preference in a two-choice assay. Behavioural results obtained with this assay indicate that gut NPF is important for the mating-induced increase in preference for yeast (Fig. 2i). Together, these data indicate that NPF from the midgut is involved in promoting yeast intake in mated females, an effect triggered by SP signalling^6^. Consistent with this notion, females with EEC-specific *NPF* knockdown mated to SP^+^ males displayed a consumption pattern similar to that of control females mated to *SP*-mutant males: both consumed more sugar and less protein than control females mated to SP^+^ males (Fig. 3a). Control females mated to *SP*-mutant males showed a yeast-preference phenotype that was intermediate between those of virgin females and females mated to SP^+^ males, as previously reported^6^. Females with EEC-specific *NPF* knockdown mated to SP^+^ males displayed lower yeast preference than control females mated to SP^+^ males, and their yeast preference was not significantly different from that of virgin females (Fig. 3b). Thus, females upregulate their protein intake after mating in a partially SP-dependent manner, and our results indicate that NPF from the EECs is involved in mediating this SP-induced shift in food choice, independently of juvenile hormone (Extended Data Fig. 3c), which is known to affect gut remodelling after mating^29^. We therefore rationalized that injection of NPF into virgin females should induce an increase in their yeast preference. As expected, virgin females injected with NPF peptide exhibited an increased preference for dietary yeast (Fig. 3c).

The induction of yeast preference and the stimulation of gut NPF release after mating are both triggered by SP receptor (SPR) activity in reproductive-tract Ppk^+^ neurons^6,17^. We found that mating did not significantly upregulate yeast preference in females with *SPR* knockdown in the Ppk^+^ neurons (Fig. 3d), confirming that SP/SPR signalling is important for the preference change. However, injection of NPF was still able to increase the yeast preference of these mated females (Fig. 3d), suggesting that gut NPF acts downstream of SP-SPR signalling in mated females to regulate food choice.

### Sut2 in NPF^+^ EECs regulates NPF release and food choice

**Fig. 4:**
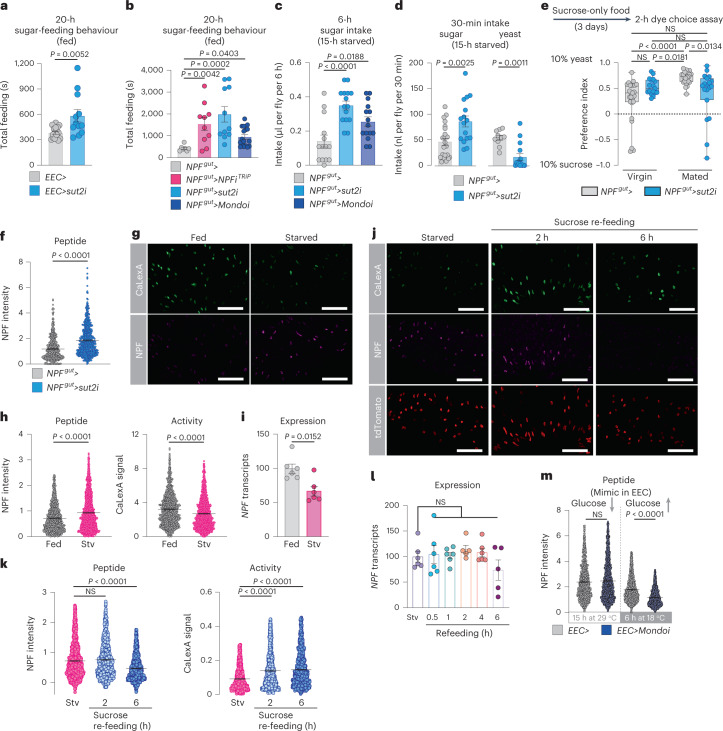
Sugar transporter 2 in the EECs regulates glucose-stimulated NPF secretion in mated females. **a**,**b**, Feeding time measured using FLIC. **a**, *n* = 16 *EEC*>, *n* = 12 *EEC*>*sut2i*. **b**, *n* = 7 *NPF*^*gut*^>, *n* = 10 *NPF*^*gut*^>*NPFi*^*TRiP*^, *n* = 11 *NPF*^*gut*^>*sut2i*, *n* = 17 *NPF*^*gut*^>*Mondoi*. **c**, Sugar consumption measured by CAFÉ assay; *n* = 14 *NPF*^*gut*^>, *n* = 15 *NPF*^*gut*^>*sut2i*, *n* = 15 *NPF*^*gut*^>*Mondoi*. **d**, Consumption of sucrose or yeast measured by dye assay. Sucrose *n* = 22 *NPF*^*gut*^>, *n* = 16 *NPF*^*gut*^>*sut2i*. Yeast *n* = 10 *NPF*^*gut*^>, *n* = 15 *NPF*^*gut*^>*sut2i*. **e**, Consumption preference measured by two-choice dye-consumption assay. Virgins, *n* = 25 *NPF*^*gut*^>, *n* = 17 *NPF*^*gut*^>*sut2i*; mated *n* = 24 *NPF*^*gut*^>, *n* = 19 *NPF*^*gut*^>*sut2i*. **f**, Midgut NPF staining with *sut2* knockdown; *n* = 571 cells from six guts for *NPF*^*gut*^>, *n* = 625 cells (five guts) for *NPF*^*gut*^>*sut2i*. **g**,**h**, Representative images of midgut NPF-cell activity (**g**), quantified in **h**; left shows *n* = 1,167 cells from eight fed guts, *n* = 1,394 cells from nine starved (stv) guts; right shows *n* = 1,209 cells from eight fed guts, *n* = 1,405 cells from nine starved guts. **i**, Midgut *NPF* expression; *n* = 6 for stv, 0.5 h, 1 h, 4 h and *n* = 5 for 2 h, 6 h, each samples of five guts. **j**, Representative images of NPF staining and cell activity in 24-h starved, 2-h sugar re-fed and 6-h sugar re-fed mated females. All measured cells are marked with tdTomato. **k**, Quantification of **j**. Eight guts per condition. Left, *n* = 1,297, 1,038 and 1,216 cells from starved, 2-h and 6-h re-fed animals. Right, *n* = 1,141, 954 and 1,151 cells from starved, 2-h re-fed and 6-h re-fed animals. **l**, Midgut *NPF* expression in mated females after re-feeding following 24-hour starvation (Stv, no re-feeding); *n* = 6 replicates of six midguts except *n* = 5 replicates for 2 and 6 h. **m**, NPF staining in mated females’ midguts after 15 hours’ knockdown of *Mondo* (29 °C inactivation of GAL80^TS^) and following subsequent 6-h derepression of Mondo (18 °C). 15 h at 29 °C: *n* = 1,595 cells/12 guts for *EEC*>, *n* = 1,840 cells/14 guts for *EEC*>*Mondoi*; 6 h at 18 °C: *n* = 1,948 cells/17 guts for *EEC*>, *n* = 2,194 cells/18 guts for *EEC*>*Mondoi*. All animals were mated females, except in **e**. Bars represent mean ± s.e.m. Box plots indicate minimum, 25th percentile, median, 75th percentile and maximum values. NS, not significant. **a**,**d** (left), Two-tailed unpaired Student’s *t*-test. **b**,**e**,**k**, One-way Kruskal–Wallis ANOVA with Dunn’s multiple-comparisons test. **c**,**l**, One-way ANOVA with Dunnett’s multiple-comparisons test. **d** (right), **f**,**h**,**i**,**m**, Two-tailed unpaired Mann–Whitney *U*-test. Scale bars, 50 μm. Source data

Given these results indicating that NPF acts as a mediator of sugar satiety, we asked whether NPF-producing EECs might be activated by sucrose ingestion. In our initial analysis of a collection of RNAi lines (Fig. 1a), we found that knockdown of *sugar transporter 2* (*sut2*), a member of the glucose-transporter class of solute carrier (SLC) proteins, in the EECs of fed mated females increased their sugar-feeding behaviour (Fig. 4a). Loss of *sut2* in NPF^+^ EECs increased animals’ sugar-feeding behaviour and sugar intake, similar to the effects observed in animals with knockdown of *NPF* itself (Fig. 4b,c and Extended Data Fig. 3d), suggesting that Sut2 might be required as part of a mechanism governing NPF production or release.

In *Drosophila* and mammals, the sugar-responsive transcription factor Mondo/ChREBP (carbohydrate-responsive-element-binding protein) contributes to many of the cellular responses to sugar^30^. To probe the molecular sugar-sensing mechanisms regulating NPF, we silenced *Mondo* specifically in NPF^+^ EECs of mated females and found that this manipulation increased both sugar-feeding behaviour and sugar intake, although not as dramatically as the loss of *NPF* or *sut2* (Fig. 4b,c). This suggests that although other mechanisms are probably involved, Mondo/ChREBP-mediated sugar sensing may contribute to NPF regulation in EECs.

Because *NPF* loss led to increased sugar intake and decreased protein feeding, we investigated whether Sut2 also affects sugar versus protein intake in mated females. We found that knockdown of *sut2* in NPF^+^ EECs led to a strongly increased intake of sucrose and a marked decrease in consumption of yeast when animals were presented with these foods separately (Fig. 4d). Similarly, when given a choice between these two foods, mated females with knockdown of *sut2* in NPF^+^ EECs displayed a reduced preference for dietary yeast (Fig. 4e). Although Sut1 has been linked to NPF secretion in virgins^21^, we did not observe significant changes in yeast preference in virgins or mated females with *sut1* knockdown in NPF^+^ EECs (Extended Data Fig. 3e). These results indicate that loss of *sut2* in NPF^+^ EECs shifts consumption towards sugar-rich food, similar to knockdown of *NPF* in these same gut cells, consistent with a role for Sut2 in regulating NPF production or release. Knockdown of *sut2* in NPF^+^ EECs led to a strong intracellular accumulation of NPF peptide, even though *NPF* transcript levels were reduced (Fig. 4f and Extended Data Fig. 3f,g), suggesting that *sut2* loss in NPF^+^ EECs leads to NPF retention and thus that Sut2 is required for normal NPF expression and release. We also found that *sut2* transcript levels in the entire midgut were strongly reduced by knockdown targeted only at the NPF^+^ gut endocrine cells (Extended Data Fig. 3g), demonstrating that *sut2* is predominantly expressed in NPF^+^ EECs.

To assess more directly whether sugar regulates NPF^+^ EECs, we exposed mated females to different nutritional conditions and observed their calcium-signalling history, using the CaLexA reporter system, in which green fluorescent protein (GFP) expression reflects calcium signalling^31^. After 24 hours of starvation, we observed decreased calcium-induced GFP signal and increased NPF peptide staining in the NPF^+^ EECs when measured on a per-cell basis, even though *NPF* transcript levels were reduced (Fig. 4g–i), indicating NPF retention. Although these measures were higher in starved animals on a per-cell basis (Fig. 4g,h), they were not significantly altered when analyzed on a one-mean-per-gut basis (Extended Data Fig. 3h). This might possibly reflect the inhibition of only a subpopulation of the NPF^+^ EECs by starvation, which could be masked by averaging all the cells, or that starvation longer than 24 hours is required for strong inhibition, as also found by a recent report showing that NPF release is reduced after 48 hours’ starvation^21^. Re-feeding with sucrose after starvation elicited a strong increase in calcium signalling within 2 hours in the NPF^+^ EECs, associated with a decrease in NPF peptide staining within 6 hours of re-feeding (Fig. 4j,k and Extended Data Fig. 3i), as also reported by an independent study^21^. Since midgut *NPF* transcript levels were unaltered under these conditions (Fig. 4l), these results indicate that midgut NPF^+^ cells are activated by dietary sugar, leading to their secretion of NPF peptide.

We then used genetic methods to mimic sugar sensing occurring in the EECs following a meal. We first induced RNAi-mediated silencing of *Mondo/ChREBP* by switching flies to 29 °C to inactivate GAL80^TS^ for 15 hours, to reduce sugar sensing in the EECs. We then reactivated Mondo/ChREBP signalling, to mimic sugar-sensing occurring after a meal, by switching animals from 29 back to 18 °C to renature GAL80^TS^ and thereby inactivate the RNAi effect. Reactivation of Mondo/ChREBP signalling in the EECs caused a decrease in NPF peptide levels in these cells without altering *NPF* expression (Fig. 4m and Extended Data Fig. 3j,k), indicating increased NPF secretion, consistent with the notion that sugar sensing in the EECs is associated with NPF release. Taken together, our findings indicate that, in mated females, sugar intake leads to EEC NPF release through a process requiring glucose-transporter-family protein Sut2 and involving Mondo/ChREBP-mediated sugar sensing.

### NPF suppresses energy mobilization in adipose tissues

**Fig. 5:**
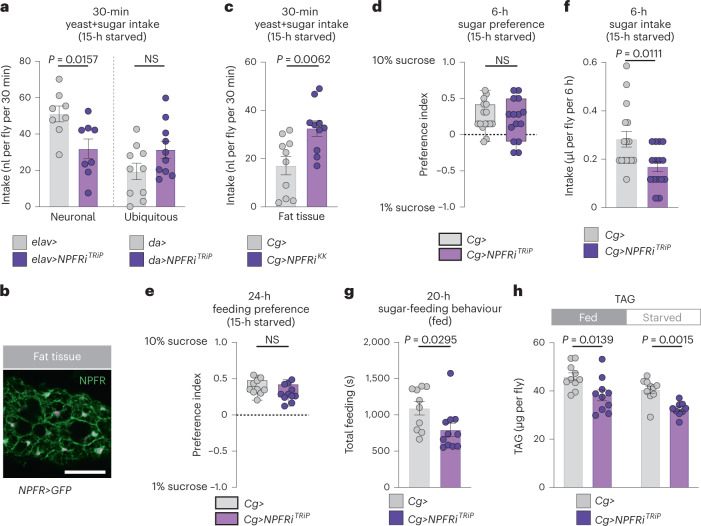
Loss of *NPFR* in the fat affects metabolism but does not increase preference for dietary sugar in mated females. **a**, Food intake measured by dye in animals with knockdown of *NPFR* in the nervous system (*elav*>) or the entire body (*da*>) measured by dye assay; *n* = 8 *elav*>, *n* = 8 *elav*>*NPFRi*^*TRiP*^, *n* = 10 *da*>, *n* = 10 *da*>*NPFRi*^*TRiP*^. **b**, Fat-body immunostaining of *NPFR*>*mCD8::GFP* reporter. Scale bar, 50 μm **c**, Food intake determined by dye assay in fat-body *NPFR*-knockdown animals; both *n* = 10. **d**, Consumption preference for 1 versus 10% sucrose, measured by CAFÉ assay; *n* = 16 *Cg*>, *n* = 15 *Cg*>*NPFRi*^*TRiP*^. **e**, Behavioural preference for interacting with 1 versus 10% dietary sucrose measured by FLIC; *n* = 10 *Cg*>, *n* = 11 *Cg*>*NPFRi*^*TRiP*^. **f**, Sucrose intake by CAFÉ assay; *n* = 16 *Cg*>, *n* = 15 *Cg*>*NPFRi*^*TRiP*^. **g**, Time spent feeding on sucrose using FLIC; *n* = 10 *Cg*>, *n* = 11 *Cg*>*NPFRi*^*TRiP*^. **h**, Whole-body TAG levels in fed and 15-hour-starved fat-body *NPFR*-knockdown animals. All *n* = 10 except *n* = 9 starved *Cg*>*NPFRi*^*TRiP*^. All animals were mated females. Bars represent mean ± s.em. Box plots indicate minimum, 25th percentile, median, 75th percentile and maximum values. NS, not significant. **a**,**c**–**e**,**h** (left), Two-tailed unpaired Student’s *t*-test. **f**–**h** (right), Two-tailed unpaired Mann–Whitney *U*-test. Source data

We then asked which target tissues might be involved in NPF-mediated appetite regulation by knocking down the *NPF receptor* (*NPFR*). Whereas EEC-specific *NPF* knockdown induced overfeeding (Fig. 1d), adult females with neuronal knockdown of *NPFR* driven by *elav-GAL4* (*elav*>) showed decreased food intake (Fig. 5a), consistent with previous reports that neuronal NPF/NPFR signalling promotes feeding^32^ and indicating that other tissues mediate the downregulation of sugar intake induced by gut NPF. Global knockdown of *NPFR* driven by *daughterless-GAL4* (*da*>) led to a feeding phenotype intermediate between those observed with gut or neuronal *NPF* signalling loss (Fig. 5a), probably reflecting opposing effects on feeding of NPF signalling within different organs.

**Extended Data Fig. 4:**
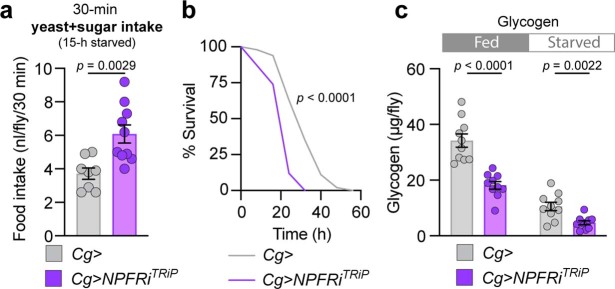
Knockdown of *NPFR* in the fat body leads to increased food intake after starvation, reduced starvation resistance, and lower glycogen stores. (**a**) Food intake measured by dye assay (medium containing 9% sugar and 8% yeast); n = 8 *Cg* > , n = 10 *Cg* > *NPFRi*^*TRiP*^. (**b**) Survival during starvation (n = 100 *Cg* > , n = 50 *Cg* > *NPFRi*^*TRiP*^). (**c**) Glycogen levels in fed and 15-hour-starved females; all n = 10. All animals were mated females. Bars represent mean±SEM. ns, non-significant. **a, c:** Two-tailed unpaired Student’s t test. **b:** Gehan-Breslow-Wilcoxon test. Source data

To assess receptor expression in other target tissues, we used a CRISPR-mediated knock-in of *T2A::GAL4* into the native *NPFR* locus (*NPFR::T2A::GAL4*, hereafter *NPFR*>)^33^ to express *UAS-mCD8::GFP*. We observed reporter expression in the fat body (Fig. 5b), a tissue analogous to adipose tissue and liver in mammals. Although fat-body-specific *NPFR* knockdown driven by *Cg-GAL4* (*Cg>*) in adult females did lead to increased short-term intake of food containing both sugar and yeast, it did not increase preference for sugar (Fig. 5c–e and Extended Data Fig. 4a). Indeed, suppression of *NPFR* in the fat body led to decreased sucrose intake and sugar-feeding behaviour (Fig. 5f,g). Thus, fat-body NPFR signalling does not appear to underlie the specific feeding phenotypes observed with gut *NPF* loss. We next asked whether fat-body NPFR mediates the effects of gut NPF on metabolism. Like animals with gut-specific *NPF* knockdown, fat-body *NPFR* knockdown animals were more sensitive to starvation and displayed metabolic phenotypes similar to those seen with loss of gut *NPF* (Fig. 5h and Extended Data Fig. 4b,c). These findings indicate that gut-derived NPF acts on NPFR in the fat body as part of a metabolic pathway that maintains energy homeostasis.

### NPF regulates food choice through glucagon-like signalling

**Extended Data Fig. 5:**
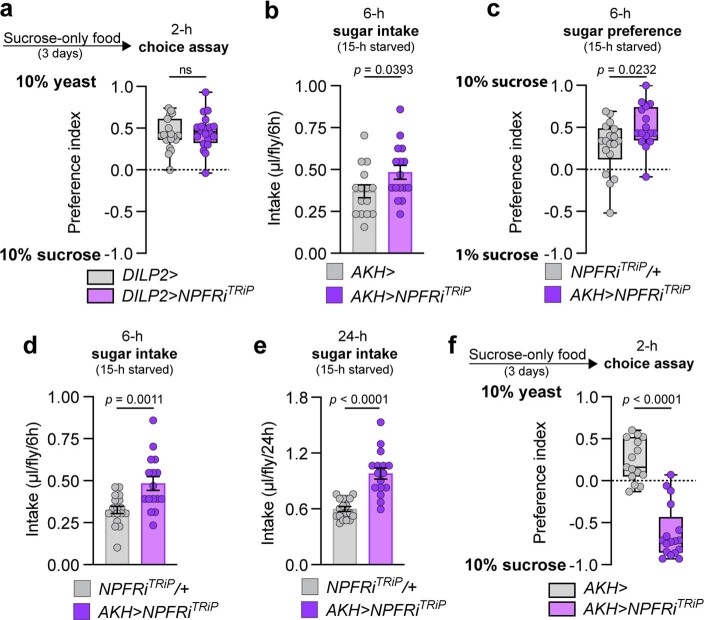
Knockdown of *NPFR* in the AKH-producing cells leads to increased preference for and intake of dietary sugar. (**a**) Consumption preference using two-choice dye assay; n = 19 *DILP2* > , n = 20 *DILP2* > *NPFRi*^*TRiP*^. (**b**) Consumption of 10% sucrose measured by CAFÉ assay; n = 15 *AKH* > , n = 16 *AKH* > *NPFRi*^*TRiP*^. (**c**) Consumption preference for 1% *vs*. 10% sucrose solution measured by CAFÉ assay; n = 18 *NPFRi*^*TRiP*^*/+*, n = 16 *AKH* > *NPFRi*^*TRiP*^. (**d**,**e**) Consumption of 10% sucrose measured over 6 hours and 24 hours by CAFÉ assay; n = 18 *NPFRi*^*TRiP*^*/+*, n = 16 *AKH* > *NPFRi*^*TRiP*^. (**f**) Consumption preference using two-choice dye assay; n = 15 *AKH* > , n = 17 *AKH* > *NPFRi*^*TRiP*^. All animals were mated females. Bars represent mean±SEM. Box plots indicate minimum, 25^th^ percentile, median, 75^th^ percentile, and maximum values. ns, non-significant. **a, d, e, f:** Two-tailed unpaired Mann-Whitney U test. **b, c:** Two-tailed unpaired Student’s t test. Source data

Our experiments indicate that gut NPF signalling regulates sugar appetite via tissues other than the CNS and fat body. In *Drosophila*, the brain cells that produce insulin express NPFR^21^ and these cells also regulate aspects of feeding and satiety^34^. However, knockdown of *NPFR* in the insulin-producing cells (IPCs) did not change preference for yeast versus sugar in mated females (Extended Data Fig. 5a), suggesting that gut-derived NPF does not act through insulin to modulate preference for dietary sugar and protein.

**Fig. 6:**
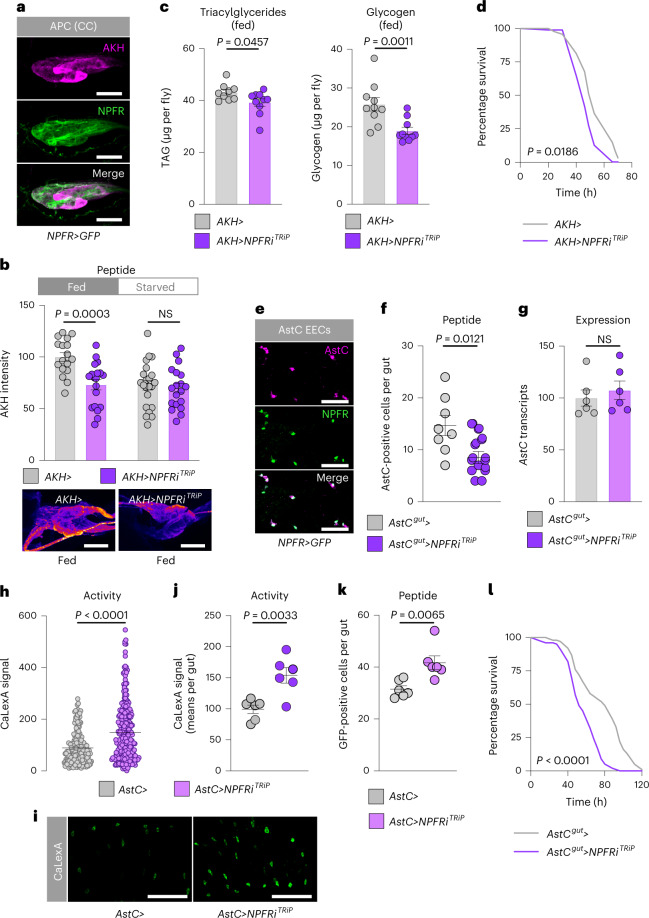
NPFR regulates metabolism through inhibition of AKH signalling in mated females. **a**, Immunohistochemistry of APCs shows *NPFR*>*GFP* reporter expression in APCs of mated females. Scale bars 20 μm. **b**, Quantification of AKH levels within the APCs of fed and 15-hour-starved mated females with and without *NPFR* knockdown in these cells; *n* = 17 fed *AKH*>, *n* = 19 fed *AKH*>*NPFRi*^*TRiP*^, *n* = 22 starved *AKH*>, *n* = 19 starved *AKH*>*NPFRi*^*TRiP*^. Representative images are shown below. Scale bars 20 μm. **c**, Metabolite levels in fed mated females. TAG *n* = 9 *AKH*>, *n* = 10 *AKH*>*NPFRi*^*TRiP*^; glycogen *n* = 10 *AKH*>, *n* = 10 *AKH*>*NPFRi*^*TRiP*^. **d**, Survival during starvation of mated females; *n* = 96 *AKH*>, *n* = 96 *AKH*>*NPFRi*^*TRiP*^. **e**, Immunohistochemistry of guts from mated female *NPFR*>*GFP* flies showing expression of *NPFR* reporter in AstC^+^ cells. Scale bars, 25 μm. **f**, Quantification of the number of midgut cells per gut that showed detectable AstC staining with and without *NPFR* knockdown in AstC^+^ EECs of fed mated females; *n* = 8 *AstC*^*gut*^> guts, *n* = 11 *AstC*^*gut*^>*NPFRi*^*TRiP*^. **g**, Midgut *AstC* transcript levels in fed mated females; each *n* = 6. **h**–**k**, AstC^+^ EEC-cell activity levels (**h**) with representative images (**i**) quantified on a per-gut basis (**j**) and the number of AstC^+^ EEC cells showing detectable GFP (**k**), measured by calcium-reporter system (*AstC*>*LexA::NFAT::VP16; LexAop-GFP*, denoted by *AstC*> in the figure) with or without *NPFR* knockdown in the AstC^+^ EECs in the midgut. **h**, *n* = 182 *AstC*> cells, *n* = 255 *AstC*>*NPFR* cells; **j**, each *n* = 6 guts; **k**, each *n* = 6 guts. Scale bars, 50 μm. **l**, Survival during starvation of mated females; *n* = 153 *AstC*^*gut*^> animals, *n* = 198 *AstC*^*gut*^>*NPFRi*^*TRiP*^. All animals were mated females. Bars represent mean ± s.e.m. Box plots indicate minimum, 25th percentile, median, 75th percentile and maximum values. NS, not ignificant. **b**,**c** (left), **f**,**g**,**j**, Two-tailed unpaired Student’s *t*-test. **d**,**l**, Gehan–Breslow–Wilcoxon test. **c** (right), **h**,**k**, Two-tailed unpaired Mann–Whitney *U*-test. Source data

To identify the tissue mediating this effect, we examined *NPFR* expression in other tissues, which revealed expression of the receptor in the cells producing the glucagon-like factor AKH (Fig. 6a). AKH is released from the AKH-producing cells (APCs) during starvation and acts through its receptor, AkhR, on the fat body to promote the mobilization of stored energy, and it is also thought to act as a hunger signal to drive feeding behaviours^18,31,35–37^. However, whether AKH regulates sugar- or protein-specific feeding is unknown. We proposed that gut-derived NPF, released in response to sugar feeding, might suppress AKH release from the APCs in the fed state. Consistent with a recent report^21^, we found that knocking down *NPFR* in the APCs using *AKH-GAL4* (*AKH*>) resulted in decreased AKH peptide levels within these cells in fed mated females (Fig. 6b), indicating that NPFR is required to suppress AKH release when the animal has ingested food. AKH promotes lipid and glycogen breakdown, and we therefore tested whether NPFR activity in the APCs regulates metabolism. As with knockdown of *NPF* in the midgut, adult females with knockdown of *NPFR* in the APCs showed reduced TAG and glycogen levels and increased susceptibility to starvation, as also reported recently^21^ and consistent with an increase in AKH signalling (Fig. 6c,d).

We recently reported that the peptide hormone AstC released by midgut EECs promotes AKH release during starvation conditions^18^. We wondered whether NPF signalling might also inhibit AstC release from the midgut to suppress the activation of the AKH axis at multiple hierarchical levels. We found expression of NPFR reporter in AstC^+^ EECs (Fig. 6e), consistent with single-cell RNA-sequencing data^38^. We silenced *NPFR* expression specifically in AstC^+^ EECs using *AstC-GAL4* (*AstC*>) with pan-neuronal GAL80 (*R57C10-GAL80*, *AstC*>–*AstC*^*gut*^> hereafter) to suppress nervous system GAL4 activity. This did not alter *AstC* expression, but it did lead to a reduction in the number of cells containing detectable AstC peptide (Fig. 6f,g), suggesting that EEC loss of *NPFR* cell-autonomously increases AstC release. Knockdown of *NPFR* with *AstC*^*gut*^> led to an increased number of active midgut AstC cells and to higher overall calcium-reporter activity (Fig. 6h–k), indicating that *NPFR* knockdown promotes AstC^+^ EEC activation. This indicates that the decreased number of AstC immune-positive cells in adult females with *NPFR* knockdown in the AstC^+^ EECs (Fig. 6f) is due to increased release of AstC peptide, which would be expected to promote AKH release, leading to more rapid depletion of energy stores and therefore reduced capacity to survive starvation^18^. Consistent with this, *NPFR* knockdown targeted to the AstC^+^ EECs led to a clear reduction in the capacity of these animals to survive starvation (Fig. 6l). Taken together, our results indicate that in response to sugar intake, gut-derived NPF inhibits the AKH axis at three levels: blocking the release of adipokineticotropic AstC from midgut EECs, blocking the release of AKH itself from the APCs and counteracting AKH’s effects on the fat body.

**Fig. 7:**
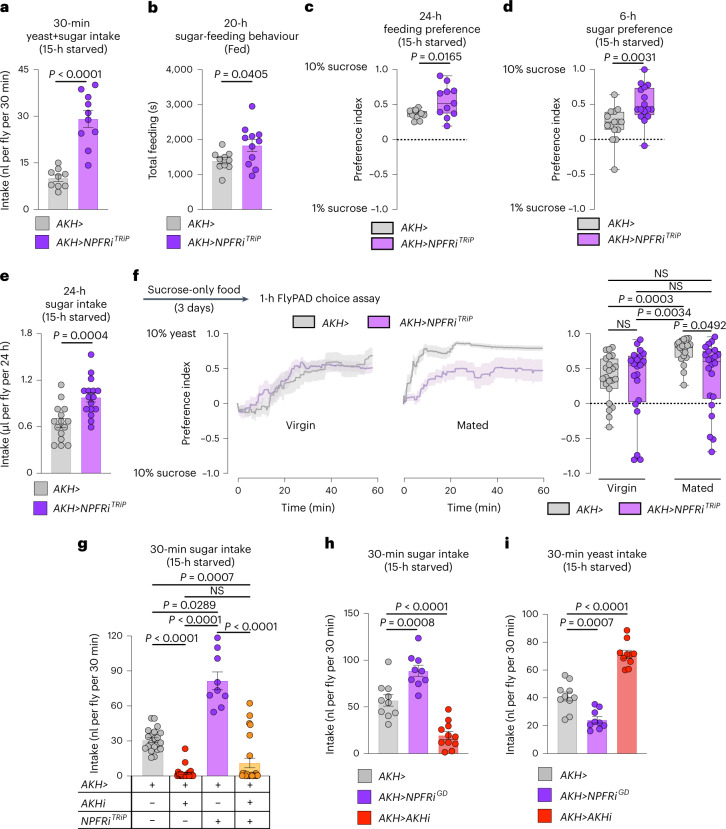
Loss of *NPFR* in the APCs phenocopies the gut *NPF*-loss feeding phenotypes in mated females. **a**, Food intake measured by dye assay; *n* = 9 *AKH*>, *n* = 10 *AKH*>*NPFRi*^*TRiP*^. **b**, Time spent feeding on sucrose measured by FLIC; *n* = 10 *AKH*>, *n* = 11 *AKH*>*NPFRi*^*TRiP*^. **c**, Behavioural preference for 1 versus 10% sugar solution measured by FLIC; both *n* = 11. **d**, Consumption preference for 1 versus 10% sucrose measured by CAFÉ assay; *n* = 15 *AKH*>, *n* = 16 *AKH*>*NPFRi*^*TRiP*^. **e**, Sucrose consumption measured by CAFÉ assay; *n* = 15 *AKH*>, *n* = 16 *AKH*>*NPFRi*^*TRiP*^. **f**, Behavioural preference using flyPAD. Lines represent means, and shading indicates s.e.m.; virgins *n* = 23 *AKH*>, *n* = 21 *AKH*>*NPFRi*^*TRiP*^; mated *n* = 19 *AKH*>, *n* = 22 *AKH*>*NPFRi*^*TRiP*^. **g**, Rescue of sugar overconsumption induced by *NPFR* knockdown in the APCs through simultaneous *AKH* knockdown, by dye assay; *n* = 21 *AKH*>, *n* = 24 *AKH*>*AKHi*^*KK*^, *n* = 9 *AKH*>*NPFRi*^*TRiP*^, *n* = 24 *AKH*>*AKHi*^*KK*^*, NPFRi*^*TRiP*^. **h**,**i**, Sucrose (**h**) and yeast (**i**) intake measured over 30 min by dye assay. **h**, *n* = 10 *AKH*>, *n* = 9 *AKH*>*NPFRi*^*GD*^, *n* = 11 *AKH*>*AKHi*^*KK*^. **i**, *n* = 10 *AKH*>, *n* = 9 *AKH*>*NPFRi*^*GD*^, *n* = 10 *AKH*>*AKHi*^*KK*^. All animals were mated females, except in **f**. Bars represent mean ± s.e.m. Box plots indicate minimum, 25th percentile, median, 75th percentile and maximum values. NS, not significant. **a**–**d**, Two-tailed unpaired Student’s *t*-test. **e**, Two-tailed unpaired Mann–Whitney *U-*test. **f**,**g**, One-way Kruskal–Wallis ANOVA with Dunn’s multiple-comparisons test. **h**,**i**, One-way ANOVA with Dunnett’s multiple-comparisons test. Source data

*NPFR* knockdown in the APCs was recently linked to increased consumption of food in virgin females^21^, an effect we confirmed in mated females (Fig. 7a). Next, we tested whether NPF regulates sugar- versus protein-specific feeding through NPFR in the APCs. Mated females with APC-specific *NPFR* knockdown exhibited elevated sugar-directed feeding behaviour, sugar consumption and preference for dietary sugar (Fig. 7b–e and Extended Data Fig. 5b–e), similar to animals with loss of *NPF* in the midgut. Next, we examined whether NPFR in the APCs might be involved in promoting yeast preference in mated females. Indeed, whereas APC knockdown of *NPFR* in virgin females did not detectably alter feeding preference, this manipulation had a strong effect in mated females (Fig. 7f and Extended Data Fig. 5f), similar to animals with knockdown of *NPF* in the gut, indicating that NPFR in the APCs is an important element for promoting consumption of protein-rich food in mated females. To determine whether AKH mediates the effects of *NPFR* loss on feeding, we examined the ability of *AKH* knockdown to rescue *NPFR-RNAi*’s sugar-overeating phenotype. We found that knockdown of *AKH* completely abolished this effect (Fig. 7g), suggesting that AKH is the primary factor mediating the feeding effects of NPFR signalling. Consistent with NPFR’s suppression of AKH release, *AKH* loss and *NPFR* knockdown induced opposite effects on sugar intake and yeast consumption (Fig. 7h,i). Together, our results indicate that, in mated females, gut-derived NPF acts on the APCs via NPFR to inhibit AKH release after a sugar-rich meal, to suppress further sugar feeding while promoting protein intake.

### AKH regulates appetites for sugar and protein-rich food

**Fig. 8:**
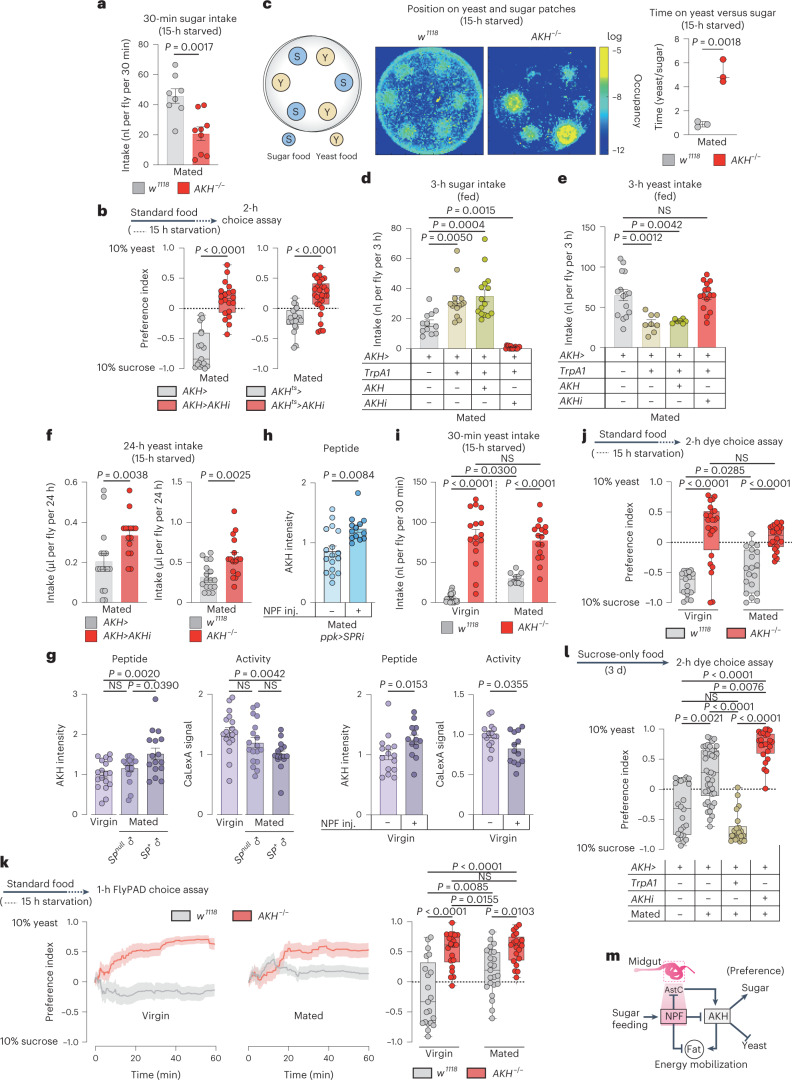
AKH promotes sugar feeding and suppresses protein intake in mated females. **a**, Sugar intake by dye assay. *n* = 8 *w*^*1118*^, *n* = 9 *AKH*^−/−^. **b**, Consumption preference, *n* = 17 *AKH*>, *n* = 20 *AKH*>*AKHi*^*KK*^, *n* = 20 *AKH*^*ts*^>, *n* = 28 *AKH*^*ts*^>*AKHi*^*KK*^. **c**, Heat map of 30 min tracking of 12 females per genotype. Ratio of time spent on yeast versus sugar patches, *n* = 3. **d**,**e**, Sugar and yeast intake using dye assay, at 29 °C for TrpA1 activation. **d**, *n* = 12 *AKH*>, *n* = 14 *AKH*>*TrpA1*, *n* = 15 *AKH*>*TrpA1*+*AKH*, *n* = 15 *AKH*>*TrpA1*+*AKHi*. **e**, *n* = 15 *AKH*>, *n* = 8 *AKH*>*TrpA1*, *n* = 7 *AKH*>*TrpA1*+*AKH*, *n* = 15 *AKH*>*TrpA1*+*AKHi*. **f**, Yeast intake measured by CAFÉ assay. *n* = 6 *AKH*>, *n* = 15 *AKH*>*AKHi*^*KK*^, *n* = 17 *w*^*1118*^, *n* = 16 *AKH*^−/−^. **g**, APCs staining and cell activity, left two panels show *n* = APCs from 18 *w*^*1118*^ virgins, *n* = 18 *w*^*1118*^ females mated to *SP*-deficient males (*SP*^*0*^/*Df(3L)delta130*), *n* = 16 *w*^*1118*^ females mated to *SP*^*+*^ males. Right two panels show *n* = 15 *w*^*1118*^ virgins without NPF injection, *n* = 13 *w*^*1118*^ virgins with NPF injection. **h**, AKH staining intensity in mated females with *SPR* knockdown in the *ppk*^*+*^ neurons with or without NPF injection, *n* = 17 *ppk*>*SPRi* mated females, *n* = 13 *ppk*>*SPRi* mated females with NPF injection. **i**, Yeast consumption measured by dye assay, *n* = 18 *w*^*1118*^ virgins, *n* = 16 *AKH*^−/−^ virgins, *n* = 10 mated *w*^*1118*^ females, *n* = 16 mated *AKH*^−/−^ females. **j**, Consumption preference using the two-choice dye assay; *n* = 17 *w*^*1118*^ virgins, *n* = 24 *AKH*^−/−^ virgins, *n* = 19 mated *w*^*1118*^ females, *n* = 22 mated *AKH*^−/−^ females. **k**, Cumulative behavioural preference using flyPAD. Lines represent means, and shading indicates s.e.m., *n* = 21 *w*^*1118*^ virgins, *n* = 22 *AKH*^−/−^ virgins, *n* = 23 mated *w*^*1118*^ females, *n* = 20 mated *AKH*^−/−^ females. **l**, Preference measured using the two-choice dye assay at 29 °C for TrpA1 activation, *n* = 23 *AKH*> virgins, *n* = 42 *AKH*>, *n* = 22 *AKH*>*TrpA1*, *n* = 24 *AKH*>*AKHi* mated females. **m**, A model of the NPF-AKH axis. Bars represent mean ± s.e.m. Box plots indicate minimum, 25th percentile, media, 75th percentile and maximum. Inj., injection; NS, not significant. **a**,**b** (right), **c**,**f**, Two-tailed unpaired Student’s *t*-test. **b** (left), **g** (right), **h**, Two-tailed unpaired Mann–Whitney *U*-test. **d**,**e**,**g** (left), **i**,**k**, One-way ANOVA with Tukey’s post hoc test. **j**, One-way ANOVA with Dunnett’s multiple comparison test or Kruskal–Wallis with Dunn’s multiple-comparisons test. **l**, One-way Kruskal–Wallis ANOVA with Dunn’s multiple-comparisons test. Source data

**Extended Data Fig. 6:**
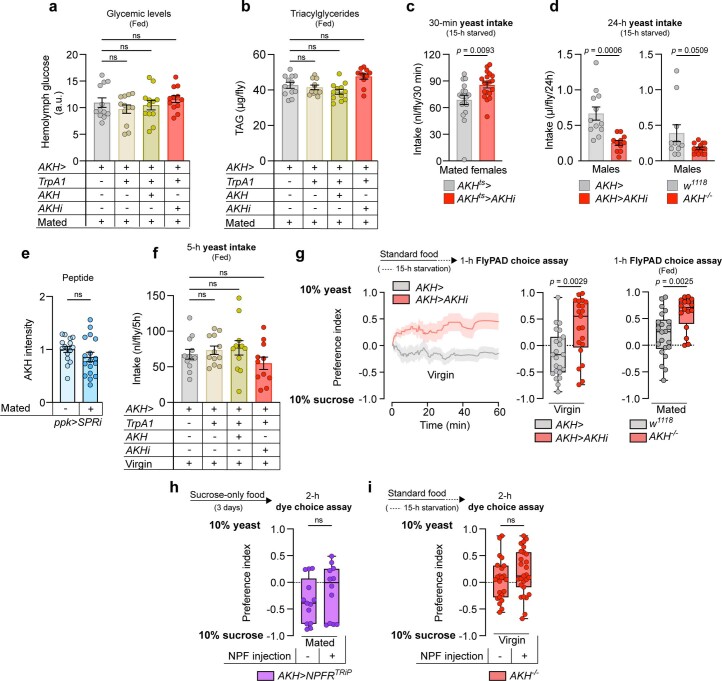
Alteration of AKH signaling leads to increased yeast intake in females, not males, without metabolic effects, indicating sexual dimorphism in AKH effects on feeding preferences. (**a**,**b**). Hemolymph glucose and TAG levels in mated females at 29 °C for TrpA1 activation; (a) all n = 12, (b) n = 12 *AKH* > , n = 11 *AKH* > *TrpA1*, n = 12 *AKH* > *TrpA1* + *AKH*, n = 11 *AKH* > *TrpA1* + *AKHi*. (**c**) Yeast consumption measured by dye assay in mated female flies; n = 18 *AKH*^*ts*^ > , n = 19 *AKH*^*ts*^ > *AKHi*^*KK*^. (**d**) Yeast intake of males measured over 24 hours by CAFÉ assay. Left panel, *AKH* knockdown; right panel, *AKH* mutant; n = 12 *AKH* > , n = 11 *AKH* > *AKHi*^*KK*^, n = 11 *w*^*1118*^, n = 15 *AKH*^-/-^. (**e**) AKH staining intensity in virgin and mated females with *SPR* knockdown in the *ppk*^*+*^ neurons; n = 16 *ppk* > *SPRi* virgins, n = 17 *ppk* > *SPRi* mated females. Note that animals were injected with hemolymph-like buffer without NPF peptide. (**f**) Yeast consumption measured in fed virgin females using dye assay, at 29 °C for TrpA1 activation; n = 12 *AKH* > , n = 12 *AKH* > *TrpA1*, n = 12 *AKH* > *TrpA1* + *AKH*, n = 11 *AKH* > *TrpA1* + *AKHi*). (**g**) Cumulative behavioural preference of virgin females after 15 hours of starvation or of fed mated females using flyPAD. Lines represent the mean and shading indicates SEM. Virgins: n = 23 *AKH* > , n = 21 *AKH* > *AKHi*^*KK*^. Mated females: n = 22 *w*^*1118*^, n = 16 *AKH*^*-/-*^. (**h**,**i**) Injection of NPF peptide into the hemolymph of 3-days-yeast-deprived mated females with *NPFR* knockdown in the APCs (h) or 15-h starved *AKH* mutant virgin females (i) using two-choice dye assay; n = 13 *AKH* > *NPFRi*^*TRiP*^, n = 12 *AKH* > *NPFRi*^*TRiP*^ with NPF injection, n = 23 *AKH*^*-/-*^, n = 27 *AKH*^*-/-*^ with NPF injection. Bars represent mean±SEM. Box plots indicate minimum, 25^th^ percentile, median, 75^th^ percentile, and maximum values. ns, non-significant. **a, b, f:** One-way ANOVA with Tukey’s multiple-comparisons test. **c, e, g, h, i:** Two-tailed unpaired Mann-Whitney U test. **d:** Two-tailed unpaired Student’s t test. Source data

AKH is described as a generic hunger hormone released during nutritional deprivation^18,35,39^. However, our findings indicate that AKH regulates food choice. To confirm this effect, we examined the feeding behaviour of mated *AKH* mutant females and found that these animals exhibited significantly reduced sugar intake (Fig. 8a), suggesting that AKH promotes sugar preference. On this basis, this we expected an increase in yeast preference with loss of *AKH*, so we used assay conditions in which animals do not normally exhibit strong yeast preference: 15-hour starvation rather than 3-day yeast deprivation. Loss of *AKH*, including adult-restricted APC-specific knockdown (with *GAL80*^*TS*^*; AKH*>, together referred to as *AKH*^*ts*^>), led to a striking shift in preference towards yeast using the two-choice dye assay in mated females (Fig. 8b). Consistent with their increased intake of and preference for yeast food, mated *AKH* mutant females displayed a strong increase in the amount of time spent exploring patches of yeast food compared to sugar patches (Fig. 8c). This further supports a role for AKH in controlling feeding decisions, biasing behaviour towards sugar intake. Consistent with this, activation of the APCs to induce AKH release caused increased sugar intake while decreasing yeast intake, effects that were blocked by simultaneous *AKH* knockdown (Fig. 8d,e), indicating that they were mediated by AKH. Circulating sugar levels and whole-body TAG levels were not altered (Extended Data Fig. 6a,b), suggesting that the observed AKH-induced feeding phenotypes are direct effects that precede detectable metabolic changes. Together, these findings indicate that AKH is a hormone that controls selective feeding decisions by increasing appetite for sugar and reducing intake of protein food.

AKH has recently emerged as a key factor in sex-specific metabolic regulation^40^. Unlike mated females, in which *AKH* loss enhanced yeast intake, males lacking *AKH* exhibited a decrease in yeast intake, indicating that AKH plays a sexually dimorphic role in feeding decisions (Fig. 8f and Extended Data Fig. 6c,d). Together these findings indicate that, in mated females, gut-derived NPF inhibits AKH secretion, and this inhibition suppresses sugar appetite and increases the consumption of protein-rich food. In this scenario, increased AKH signalling in virgins promotes preference for dietary sugar. We therefore examined whether mating reduces AKH release. Mating increased AKH peptide levels and reduced the calcium activity of the APCs, indicating repression of AKH release, and the effect on AKH was dependent on SP signalling (Fig. 8g). These findings suggest that SP signalling, through activation of gut NPF, contributes to the suppression of AKH release after mating. Injecting NPF peptide into virgin females increased AKH peptide levels within the APCs and reduced their calcium activity, indicating that NPF is sufficient to repress AKH secretion (Fig. 8g). Mating did not lead to AKH retention in females with knockdown of *SPR* in the Ppk^+^ neurons (Extended Data Fig. 6e), further indicating that SP-SPR signalling is required to repress AKH signalling after mating. To test whether NPF functions downstream of SP-SPR signalling in the AKH-regulatory hierarchy, we injected NPF into mated females with reduced SP-SPR signalling (*ppk*>*SPR-RNAi*) and found that this led to increased AKH peptide levels in the APCs (Fig. 8h). This suggests that NPF is sufficient downstream of the SP pathway to repress AKH signalling after mating, which increases yeast intake. We therefore conjectured that loss of *AKH* would increase yeast intake in virgin females, whereas increasing AKH signalling in virgin females would have little effect on feeding behaviour. To assess the effect of loss of *AKH*, we examined behaviour under starved conditions, under which AKH signalling is normally high. As expected, we found that while control females increased their yeast consumption in response to mating, similarly conditioned *AKH* mutant virgins displayed a striking overconsumption of yeast food that was not significantly altered by mating (Fig. 8i). Activation of the APCs to induce AKH release in the fed state, in which AKH signalling is generally lower, did not alter yeast intake in fed virgin females (Extended Data Fig. 6f), presumably because of the already higher AKH signalling in the virgin state, whereas as mentioned above this treatment did reduce the yeast intake of fed mated females (Fig. 8e). Consistent with these findings, virgin *AKH* mutant females exhibited a strong preference for yeast food that did not increase significantly in response to mating as it did in control females (Fig. 8j,k and Extended Data Fig. 6g). Together, this suggests that, in mated females, NPF acts through NPFR in the APCs to repress AKH signalling, which increases yeast intake. Consistent with this, we found that inducing AKH release was sufficient to block the high yeast preference exhibited by mated females after 3 days of yeast deprivation (Fig. 8l). To demonstrate that NPF regulates food choice via AKH, we injected NPF into mated females with APC-specific *NPFR* knockdown. This manipulation did not increase yeast preference after 3 days’ yeast deprivation (Extended Data Fig. 6h), suggesting that NPFR in the APCs is required for NPF to promote yeast preference. Furthermore, injection of NPF into *AKH* mutant virgin females also did not alter sugar versus yeast preference, indicating that AKH is required for mediating the effects of NPF-NPFR on food preference (Extended Data Fig. 6i). We propose that sugar-induced AKH-repressive NPF signalling from the gut constitutes a hormonal axis involved in suppressing sugar appetite and promoting intake of protein-rich food in mated females (Fig. 8m).

## Discussion

To maintain nutritional homeostasis, animals need to match their ingestion of specific nutrients to their needs. This is achieved by modulating appetite towards the specific nutrients needed. A number of factors, including gut hormones, that regulate food consumption have been identified in both flies and mammals, and reports have also described central brain mechanisms that induce ingestion of protein food in response to amino-acid deprivation, that sense amino acids and promote food consumption and that reject food lacking essential amino acids^1,6,12,41,42^. However, little is known about the hormonal mechanisms that regulate nutrient-specific appetite, and gut hormones that regulate selective food intake are completely unknown. Our findings indicate that, in mated female *Drosophila*, gut-derived NPF is a selective driver of sugar satiety and protein consumption, providing a basis for understanding these mechanisms. Hormone-based therapies that inhibit appetite offer promising new directions for weight-loss treatment^14^. For example, Fibroblast growth factor 21 (FGF21) is a liver-derived hormone that promotes protein consumption, and it is emerging as a promising target for metabolic disorders^43^. Uncovering appetite-regulatory hormones such as gut-derived NPF that specifically inhibit sugar consumption while promoting the intake of protein-rich foods could provide effective new weight-management strategies by promoting healthier food choices.

The SLC2-family sugar transporter Sut2 is the closest *Drosophila* homologue of human SLC2A7 (GLUT7), a transporter expressed mainly in the intestine whose function is poorly defined^44^. In flies, GLUT1 is important for Bursicon secretion from the EECs, and Sut1, another SLC2-family sugar transporter protein, was recently shown to be involved in midgut NPF release in virgin females^20,21^. Our results implicate Sut2 in the release of NPF from EECs in mated females and thus link it to the mechanism by which NPF-mediated gut signalling controls feeding decisions. This indicates that both Sut1 and Sut2 sugar transporters are involved in glucose-stimulated NPF secretion from the gut. In mammals, several mechanisms also regulate glucose-stimulated GLP-1 secretion from intestinal endocrine cells, which involves sodium-glucose cotransporter 1 (SGLT1), the glucose transporter GLUT2 and sweet taste receptors^45^. Targeting of these intestinal glucose-sensing mechanisms therefore has become a focus of weight-management therapies because of its potential in regulating appetite and incretin effects^46^. Future studies should investigate whether GLUT7, like its *Drosophila* homologue Sut2, affects appetite-regulatory mechanisms in the mammalian gut.

NPF is orthologous with the mammalian NPY family of gut-brain peptides, including peptide YY (PYY), pancreatic polypeptide and NPY itself, that regulate food-seeking behaviours and metabolism^47,48^. Like mammalian NPY-family hormones, *Drosophila* NPF is expressed in both the nervous system and the gut. While NPY is abundant in the nervous system and, like brain NPF, promotes food intake, PYY is mainly produced by endocrine cells of the gut as a satiety factor. Gut-expressed PYY is homologous to NPY, and both act through specific G-protein coupled receptors, called NPY receptors (NPYRs), that are orthologous with *Drosophila* NPFR^49^. Thus, in mammals, multiple NPY-family peptides from different tissues sources exert their functions on target organs through several related NPYRs, while in *Drosophila*, these functions may be regulated through the single peptide–receptor pair of NPF and NPFR^47^.

Our results indicate that gut-derived *Drosophila* NPF fulfils the function of mammalian PYY. PYY is produced by the endocrine l-cells of the gut, which, like the EECs of *Drosophila*, produce a context-dependent combination of multiple hormones^49^. The physiological role of PYY in feeding regulation has been difficult to clarify, but it is believed to act through different NPYRs on tissues including the hypothalamus and the pancreatic islets to suppress appetite. Our findings show that, in flies, NPF injection strongly reduces the intake of sugar-containing food and promotes the ingestion of protein-rich food. In humans, PYY infusion also been shown to strongly reduce food intake. Although the satiety function of human PYY has made it a prime therapeutic target for potential weight management, it is not clear whether PYY regulates nutrient-specific appetite, which would be important from a therapeutic perspective. Our results indicate that *Drosophila* gut NPF, perhaps filling the role of mammalian gut PYY, acts to mediate sugar-specific satiety, illustrating a key hormonal mechanism that underlies selective hunger by which animals adjust their intake of specific nutrients.

Feeding decisions are based on internal state and exhibit sexual dimorphism. In *Drosophila*, males and females differ in their preference for and intake of dietary sugar and protein^6^. Our findings define a complex interorgan communication system through which mating influences food choices in females. We have found that midgut NPF is involved in mediating SP-induced postmating responses in females, inhibiting sugar appetite and promoting the ingestion of protein-rich yeast food, and we have further shown that AKH is required for mediating the effects of NPF. When mated females consume dietary carbohydrates, NPF is released from the EECs and inhibits the AKH axis by directly suppressing AKH release from the APCs as well as by inhibiting the release of midgut AstC, a factor that stimulates AKH secretion^18^. Furthermore, NPF acts directly on the fat body through NPFR to inhibit energy mobilization, thereby antagonizing AKH-mediated signalling in the adipose tissue. Likewise, mammalian NPY-family peptides also regulate metabolism by direct actions on adipose tissue via NPYR^50^. Although a number of studies have demonstrated that AKH is a regulator of metabolism (reviewed in ref. ^2^), our findings uncover a key role of AKH in governing nutrient-specific feeding decisions. It is becoming clear that the APCs integrate many signals that affect AKH release^18,20,21,36,51,52^, and these signals may therefore also affect food choice. The APCs therefore seem to function as a signal-integration hub, similar to the IPCs, which receive many different inputs to control insulin production and release. AstC, Bursicon and NPF from the gut control AKH expression and secretion, indicating that multiple signals, even from the same organ, converge on the APCs. These signals presumably convey different aspects of nutritional status and may act with different dynamics to regulate AKH production and/or release, or even in a redundant manner to regulate AKH signalling. Likewise, many signals released from the fat body convey similar and seemingly redundant nutritional information to the IPCs^2,53,54^.

Recent work has also revealed a sex-specific role of AKH, with lower activity in females underlying differences in male and female metabolism^40^. Consistent with this notion, our results indicate that in mated females the midgut NPF system inhibits AKH signalling, suppressing intake of sugar-rich food. Furthermore, we recently showed that in mated females, midgut-derived AstC acts in a sex-specific manner through AKH to coordinate metabolism and food intake under nutritional stress^18^. Our work here shows that NPF also works sex-specifically to sustain physiological requirements in mated females by signalling from the gut to control AKH, suggesting that the gut-AKH axis occupies a central link in the hormonal relays underlying sex-specific regulation of physiology. A recent report showed that female germline cells modulate sugar appetite, but this effect is not induced by mating and does not affect yeast feeding^55^ as we have found here for gut NPF and AKH, suggesting that it is an independent mechanism.

How nutrient signals from the gut modulate feeding is key to understanding how nutritional needs are translated into specific feeding actions to maintain balance. We have identified a homeostatic circuit triggered by gut-derived NPF that limits sugar consumption. Similar mechanisms for sugar-induced satiety that promote protein consumption may also enable mammals to balance their intake of different nutrients with their metabolic needs. Explaining how nutrient-responsive gut hormones such as NPF affect dietary choice is important to better understand hunger and cravings for specific nutrients that may ultimately lead to obesity.

## Methods

### *Drosophila* stocks and husbandry

Flies were reared on a standard cornmeal diet (82 g l^−1^ cornmeal, 60 g l^−1^ sucrose, 34 g l^−1^ yeast, 8 g l^−1^ agar, 4.8 ml l^−1^ propionic acid and 1.6 g l^−1^ Tegosept/methyl-4-hydroxybenzoate) at 25 °C and 60% humidity with a 12-h light:12-h dark daily cycle. Flies were transferred after eclosion to an adult-optimized cornmeal-free diet (90 g l^−1^ sucrose, 80 g l^−1^ yeast, 10 g l^−1^ agar, 5 ml l^−1^ propionic acid and 15 ml l^−1^ of 10% methyl-4-hydroxybenzoate in ethanol)^23^ and aged for 4–7 d before experiments. Virgin female flies were collected within 3–5 h of eclosion, whereas mated flies were sorted by sex 1 d before experiments. Genotypes that contained temperature-sensitive *Tubulin-GAL80*^*TS*^ were raised at 18 °C and kept on adult food for 3–4 d posteclosion, after which they were incubated at 29 °C for 5 d to induce RNAi effects before experiments began. The animals were transferred to fresh food every third day. The following lines were obtained from the University of Indiana Bloomington *Drosophila* Stock Center (BDSC): *AKH-GAL4* (no. 25684); *AstC::2A::GAL4* (no. 84595)^25^; CaLexA system (no. 66542: *LexAop-CD8::GFP::2A::CD8::GFP; UAS-LexA::VP16::NFAT, LexAop-CD2::GFP/TM6B, Tb*)^56^; *Cg-GAL4* (no. 7011); *da-GAL4* (no. 55850); *elav-GAL4* (no. 458); *NPF::2A::GAL4* (no. 84671)^25^; *SP*^*0*^ mutant (no. 77892); *Tub-GAL80*^*TS*^ (no. 7108); *UAS-mCD8::GFP* (no. 5137); *UAS-NPF-RNAi*^*TRiP*^ (no. 27237); *UAS-NPFR-RNAi*^*TRiP*^ (no. 25939); *10xUAS-IVS-myr::tdTomato[su(Hw)attP8]* (no. 32223); *UAS-sut1-RNAi* (no. 65964) and *UAS-TrpA1* (no. 26263). Other lines were obtained from the Vienna *Drosophila* Resource Center: the control line *w*^*1118*^ (no. 60000, isogenic with the Vienna *Drosophila* Resource Center RNAi lines) as well as several *UAS-RNAi* lines including ones targeting *AKH* (no. 105063), *Mondo* (no. 109821), *NPF* (*NPFi*^*KK*^, no. 108772 and *NPFi*^*sh*^, no. 330277), *NPFR* (*NPFRi*^*GD*^, no. 9605), *SPR* (no. 106804) and *sut2* (no. 102028). A second *UAS-TrpA1* insertion, into *attP2*, was a kind gift from C. Wegener (University of Würzburg). *voilà-GAL4* (ref. ^57^) was kindly given by A. Scopelliti (University of Glasgow). *R57C10-GAL80-6* (refs. ^58–63^) on the X chromosome was a kind gift from R. Niwa (University of Tsukuba). *AKH* mutant^64^ and *NPFR::T2A::GAL4*^33^ were kind gifts from S. Kondo (Tokyo University of Science). *Df(3L)delta130* was a kind gift from A. von Philipsborn (Aarhus University). *UAS-LexA::VP16::NFAT; LexAop-luciferase* was a kind gift from M. Rosbash (Brandeis University). The fly lines used are listed in Supplementary Table 1. No ethical approval is needed for the use of the fruit fly *Drosophila*. For standardizing the genetic background and generating controls with proper genetic background, all GAL4 lines and GAL80 lines used this study were backcrossed for several generations to the same *w*^*1118*^ genetic background population before they were used in a final outcross with the genetic background of the RNAi lines and used as controls^18^.

### Starvation-survival assay

Flies were transferred without anaesthesia to vials containing starvation medium (1% agar in water) and kept either at 29 or 25 °C, depending on whether they carried *GAL80*^*TS*^. Forty to 150 animals, at 10–15 flies per vial, were assayed for each genotype/sex. Dead animals were counted every 4–8 h. The statistical significance of survival differences was determined by using the Kaplan–Meier log-rank survival test or Gehan–Breslow–Wilcoxon survival test in the Prism software package (GraphPad v.9).

### Feeding assays

Short-term food consumption was measured by using a spectrophotometric dye-feeding assay^65,66^, and all food intake experiments were performed during the time when animals have their morning meal (1 h after lights on; 12/12 h light/dark cycle). During the morning meal (after lights on), flies were transferred without anaesthesia to adult-optimized food containing 0.5% erioglaucine dye (brilliant blue R, FD&C Blue No. 1, Sigma-Aldrich, no. 861146) and allowed to feed for 30 min, if the flies had been 15 h starved to stimulate food intake or for 2–3 h if not. Another set of flies was fed with undyed food to measure the baseline absorbance of fly lysates. For two-choice assays, the protocol of Ribeiro and Dickson^6^ was used with some modifications. Briefly, 25 flies were lightly anaesthetized with CO_2_ before being placed into a 60-mm Petri dish with a chequerboard array of 20-μl patches of alternative diets containing either 100 g l^−1^ of sucrose and dyed red with 0.5% amaranth (Sigma no. A1016), or 100 g l^−1^ yeast (dyed with 0.5% erioglaucine) and allowed to eat for 2 h in the dark. For each genotype, 10–25 samples of 1–2 flies each were homogenized in 100 μl of phosphate buffer, pH 7.5, using a TissueLyser LT (Qiagen) bead mill with 5-mm stainless-steel beads (Qiagen, no. 69989). Homogenates were centrifuged at 16,000*g* for 5 min and 50 μl of each cleared supernatant was loaded into a 384-well plate. Sample absorbance was measured at 520 nm (amaranth) and at 629 nm (erioglaucine) on an Ensight multimode plate reader (PerkinElmer). Standard curves for erioglaucine and amaranth were used to convert absorbance values to food consumption amounts.

Long-term food intake was monitored using the CAFÉ capillary-feeding assay^24^. For one-choice consumption assays, assay tubes were constructed by inserting a 5-μl microcapillary (Hirschmann) through a hole in the lid of a 2-ml Eppendorf tube. The capillary was filled with a liquid sugar or yeast-extract medium^24^ containing 100 g l^−1^ sucrose or 100 g l^−1^ yeast extract, with 1 ml l^−1^ propionic acid and 1 g l^−1^ methyl-4-hydroxybenzoate preservatives, before the start of the experiment. For sugar-preference assays, two capillaries were inserted into each tube, one filled with 10 g l^−1^ sucrose solution and the other filled with 100 g l^−1^ sucrose solution. Individual flies were briefly anaesthetized on ice and placed into assay tubes, and the tubes were placed inside a moist chamber within a standard fly incubator. The level of the meniscus in each tube was measured at intervals. Tubes containing no flies were used as controls for evaporation; the amount of meniscus movement in these tubes was subtracted from the other measurements.

To monitor feeding behaviour, interactions with food were measured over a 20–24 h period using the FLIC assay^22^. *Drosophila* feeding monitors (DFMs) (Sable Systems) were installed in an incubator (25 or 29 °C if *GAL80*^*TS*^ was present; 70% humidity, 12/12-h light/dark cycle). Feeding wells were filled with a 10% sucrose solution, and individual flies were placed in each of the 12 chambers of the DFMs in the afternoon (after the morning meal) and left to acclimate for several hours, after which evening feeding data were recorded. The next morning, at lights on, fresh sugar solution was added to the DFMs and morning meal data were recorded. In two-choice sugar-preference experiments, half of the DFM wells were filled with 1% sugar solution and the other half with 10% sugar solution. The data were recorded using the manufacturer’s software and analyzed in R Studio using the published package, available at https://github.com/PletcherLab/FLIC_R_Code.

For flyPAD^28^ two-choice behavioural experiments, 4–7-day-old female flies, mated or virgin, were either starved for 15 h on 1% agar (to establish a low yeast-preference baseline for experiments in which a manipulation was anticipated to increase this preference) or yeast-deprived for 3 days by keeping them on medium containing 10% sucrose and 1% agar (to establish a higher baseline yeast preference, for experiments in which yeast preference was expected to decrease). Flies were briefly immobilized on ice and transferred with a brush into flyPAD behavioural arenas. They were left to acclimate for several minutes before data acquisition was started. The flies were allowed to choose between food droplets containing 1% agarose and either 10% sucrose or 10% yeast. The food was aliquoted into 1.5-ml tubes and kept frozen at −20 °C. Before each experiment, the tubes were placed into a heat block for melting at 90 °C and 3 µl of food was loaded into each well. A package created by the developer for the Bonsai data-stream processing program was used to acquire the data. Data processing was done by using the developer’s software, which is available at http://www.flypad.pt.

### Video recording of feeding behaviour

Behaviour chambers (40 mm in diameter) were coated with fluon on the top and sides to prevent flies’ walking on these surfaces. Fifteen-microlitre patches of either 10% sucrose or 10% yeast (with no dyes) were placed in a circular pattern within the arena. Twelve 15-hour-starved animals per genotype were introduced into the chamber and were allowed to acclimate in darkness for a few minutes. The behaviour chambers were placed on an infrared-light transilluminator viewed by a Basler camera, and half-hour videos were recorded at 15 Hz using an imaging setup described elsewhere^67^. Flies were tracked using the Ctrax software^68^, and locomotion data were analyzed using custom MATLAB code (Code availability statement).

### Immunohistochemistry and confocal imaging

Adult midguts, CNSs and fat bodies were dissected in cold PBS and fixed for 1 h in 4% paraformaldehyde at room temperature with agitation. Anterior parts of guts, containing APCs (CCs) were first fixed for 30 min, finely dissected and fixed for a further 30 min. Fixed tissues were quickly rinsed once with PBST (PBS with 0.1% Triton X-100, Merck no. 12298) and washed in PBST three times for 15 min each. Washed tissues were incubated in blocking solution (PBST containing 5% normal goat serum (Sigma)) for 30 minutes at room temperature and incubated with primary antibodies diluted in blocking solution overnight (or 2 d for CNS samples) at 4 °C with gentle agitation. Primary-antibody solution was removed, and the tissues were rinsed once and washed three times, 20 minutes each, with PBST. Tissues were incubated with secondary antibodies diluted in PBST overnight at 4 °C, washed three times with PBST and mounted in Vectashield mounting medium containing 4,6-diamidino-2-phenylindole (Vector Laboratories, no. H-1200) on slides treated with poly-l-lysine (Sigma, no. P8920). Tissues were scanned on a Zeiss LSM-900 confocal microscope using a 20× air objective using the Zen software package. Image analysis was carried out using the open-source program ImageJ^69^. For quantification of NPF, AstC, AKH and GFP (CaLexA reporter) staining intensity, samples to be compared were stained simultaneously using the same reagent preparations and imaged with the same settings. Relevant midgut regions (the entire NPF-expressing region or the AstC-positive region) were tiled at 20× with 10–20 *Z*-stacks of at least 100 planes separated by 1 μm. Tiled stacks were stitched into a single large stack for each gut using the Stitching function of Zeiss Zen Blue v.3.1. For quantification of NPF and CaLexA staining, a binary mask containing identified cells was created using local thresholding in ImageJ. This mask was manually curated in ImageJ by comparison with the raw image data, and incorrectly joined cells were manually resegmented. In a custom MATLAB script (Code availability statement), this mask was applied to the image data to segment out each cell. Staining intensity within each cell was summed, and the local background of each cell was removed by measuring the signal around the circumference of each cell. For AstC quantifications, stacks were *Z*-projected using the sum method. Cells were manually segmented, and their intensity was measured using ImageJ with local background subtraction. We integrated a *UAS-tdTomato* transgene into the CaLexA system to normalize calcium-dependent GFP fluorescence. Antibodies used included a rabbit antibody against the processed AKH peptide^37^, a kind gift of J. Park, University of Tennessee, 1:500; rabbit anti-NPF (Ray BioTech, no. RB-19-0001-20), 1:500; rabbit anti-AstC^70^, kindly given by J. Veenstra, University of Bordeaux and M. Zandawala, Brown University, 1:500; mouse anti-GFP (ThermoFisher, no. A11120), 1:500; rat anti-mCherry (used against tdTomato; ThermoFisher, no. M11217), 1:2,000; mouse anti-Prospero (University of Iowa Developmental Studies Hybridoma Bank, no. MR1A), 1:20; Alexa Fluor 488-conjugated goat anti-mouse (ThermoFisher, no. A32723), 1:500; Alexa Fluor 555-conjugated goat anti-rabbit (ThermoFisher, no. A32732), 1:500; Alexa Fluor 555-conjugated goat anti-rat (ThermoFisher, no. A21434), 1:500 and Alexa Fluor 405-conjugated goat anti-rabbit (ThermoFisher, no. 31556), 1:500.

### Injection and methoprene-treatment experiments

Synthetic amidated NPF peptide (SNSRPPRKNDVNTMADAYKFLQDLDTYYGDRARVRFamide) was a kind gift from F. Hauser (University of Copenhagen). Peptide was dissolved at 25 μM in a synthetic haemolymph-like buffer^71^ containing 5 mM glucose, 5 mM trehalose and 110 mM sucrose (inert osmolyte). Flies were reared at 18 °C as described above. After 4 days at 29 °C to permit GAL4 activity, flies were starved on 1% agar for 15 h or protein-deprived on sugar-agar for 3 days. Flies were immobilized on ice and 50 nl of haemolymph-like solution with or without NPF was injected into each animal at the lateral mid-thorax ventral to the wings using a Nanoject II injector (Drummond Scientific). Assuming each injected animal contained 1 μl of haemolymph, the final NPF concentration in the injected animals was increased by 1.25 μM, a level that should strongly activate NPFR (IC_50_ roughly 60 nM, ref. ^72^). Animals were allowed to recover for 30 min at 29 °C before use in dye-feeding assays as described above.

Because the juvenile hormone analogue methoprene (Sigma-Aldrich no. 33375) is not thermally stable, the working solution (0.01 µg µl^−1^ in acetone) or vehicle (pure acetone) was applied to the surface of cooled, solidified fly medium instead of being mixed into melted medium before solidification. We applied 32 µl of hormone or vehicle solution to the surface of 2 ml of fly medium in 25-mm diameter plastic vials (in total, 0.32 µg per vial), an amount that should be effective while also being well tolerated by the animals^73^. Treated media were kept at room temperature for roughly 12 h to allow acetone evaporation before flies were added.

### Metabolite measurements

Triglyceride and glycogen levels were measured using established protocols^23,74^. For each genotype, ten batches of three flies each were homogenized in PBS containing 0.05% Tween-20 (Sigma no. 1379) in a TissueLyser LT (Qiagen) bead mill with 5-mm stainless-steel beads. Glycogen was measured by hydrolysing glycogen into glucose by using amyloglucosidase (Sigma, no. A7420) followed by colorimetric glucose measurement (Sigma, no. GAGO20). TAG levels were assayed by cleaving their ester bonds using Triglyceride Reagent (Sigma, no. T2449) to obtain free glycerol, the level of which was then colorimetrically measured using the Free Glycerol Reagent (Sigma, no. F6428). For determination of circulating glucose concentration, haemolymph was extracted as described previously^23^ and glucose was measured using the colorimetric assay (Sigma, no. GAGO20). Each sample’s absorbance at 540 nm was measured in a 384-well plate using an Ensight multimode plate reader (PerkinElmer) and converted to metabolite concentrations using glycerol and glucose standard curves. Measurements are reported on a per-fly basis.

### Luciferase assay

Female guts were dissected into lysis buffer (Promega, no. E2920). For each condition, 4–7 replicates with two guts in each were homogenized in 50 µl of lysis buffer in 2-ml round-bottomed Eppendorf tubes using a TissueLyser LT (Qiagen) bead mill with 5-mm stainless-steel beads (Qiagen, no. 69989). Homogenates were centrifuged at 21,000*g* for 5 min, and the supernatant was transferred into new tubes and centrifuged a second time. Ten microlitres of the cleared supernatant were loaded into a 384-well plate, and 10 µl of Dual Glo Stop & Glo Reagent (Promega) was added. The plate was left to incubate for 15 min at room temperature to allow for the reaction to pass from the burst phase into the glow phase, after which luciferase activity was measured using the luminescence mode of an Ensight multimode plate reader (PerkinElmer).

### Transcript measurement using quantitative PCR

Six tissue replicates (each containing five CNSs, five midguts or five CC-containing anterior parts of guts) per condition or genotype were homogenized in 2-ml Eppendorf tubes containing lysis buffer with 1% beta-mercaptoethanol using a TissueLyser LT bead mill (Qiagen) and 5-mm stainless-steel beads (Qiagen no. 69989). RNA purification was performed using the NucleoSpin RNA kit (Macherey-Nagel, no. 740955) according to the manufacturer’s instructions. Complementary DNA was synthesized using the High-Capacity cDNA Synthesis kit (Applied Biosystems, no. 4368814). Quantitative PCR was done using RealQ Plus 2× Master Mix Green (Ampliqon, no. A324402) on a QuantStudio 5 (Applied Biosystems) machine. Results were normalized against the housekeeping gene *Rp49* using the delta-delta-Ct method. The oligos used are listed in Supplementary Table 2.

### Statistics

All statistics were computed using the Prism analysis package (GraphPad v.9). Starvation-survival curves were analyzed using Kaplan–Meier log-rank tests or Gehan–Breslow–Wilcoxon test. Other data were assessed for normality before comparisons were performed. For normally distributed data, pairwise comparisons were made using two-tailed unpaired Student’s *t*-tests and multiple samples were compared using one-way analysis of variance (ANOVA) with post hoc multiple-comparisons tests. Other data were compared using two-tailed unpaired Mann–Whitney *U*-tests or one-way Kruskal–Wallis ANOVA followed by multiple-comparisons tests. Bar plots show the mean plus or minus the standard error of the mean (s.e.m.). Box plots that show the median and the first and third quartile, with whiskers indicating the full range of values. No data were excluded. Sample size was chosen on the basis of similar previously published studies of *Drosophila* behaviour and metabolism^17,18,20,55^. No sample-size calculations were performed.

### Reporting summary

Further information on research design is available in the Nature Research Reporting Summary linked to this article.

## Supplementary information

Supplementary Information Supplementary Tables 1–2

Reporting Summary

## Data Availability

All data generated or analyzed during this study are available as Source Data files, which are provided with this paper.

## Source data

Source Data Fig. 1 Numerical chart data.
Source Data Fig. 2 Numerical chart data.
Source Data Fig. 3 Numerical chart data.
Source Data Fig. 4 Numerical chart data.
Source Data Fig. 5 Numerical chart data.
Source Data Fig. 6 Numerical chart data.
Source Data Fig. 7 Numerical chart data.
Source Data Fig. 8 Numerical chart data.
Source Data Extended Data Fig. 1 Numerical chart data.
Source Data Extended Data Fig. 2 Numerical chart data.
Source Data Extended Data Fig. 3 Numerical chart data.
Source Data Extended Data Fig. 4 Numerical chart data.
Source Data Extended Data Fig. 5 Numerical chart data.
Source Data Extended Data Fig. 6 Numerical chart data.

## Code availability

The custom MATLAB scripts used for image analysis and for locomotion data analysis in this study are publicly available at 10.5281/zenodo.6641933 and 10.5281/zenodo.6641957.
