# Supplementary material for: A gut-derived hormone suppresses sugar appetite and regulates food choice in Drosophila
Source: Nat Metab. 2022 Nov 7;4(11):1532–50. doi: 10.1038/s42255-022-00672-z (PMC9684077; doi:10.1038/s42255-022-00672-z)
Supplement: Supplementary file 1 — Supplementary Tables 1–2 [file 42255_2022_672_MOESM1_ESM.pdf]

nature metabolism

Article https://doi.org/10.1038/s42255-022-00672-z

A gut-derived hormone suppresses sugar appetite and regulates food choice in *Drosophila*

In the format provided by the authors and unedited

Supplementary Table 1: Fly lines used in this work

Fly line	Stock number	Source
10xUAS-IVS-myr::tdTomato[su(Hw)attP8]; CaLexA	n/a	this work
AKH^{ts}> (*Tub-GAL80*^{TS}; *AKH-GAL4*)	n/a	this work
AstC^{gut}> (*R57C10-GAL80; AstC::2A::GAL4*)	n/a	this work
EEC> (*Tub-GAL80*^{TS} *voilà-GAL4* recombined on III)	n/a	this work
NPF^{gut}> (*R57C10-GAL80; NPF::2A::GAL4*)	n/a	this work
10xUAS-IVS-myr::tdTomato[su(Hw)attP8]	32223	Bloomington *Drosophila* Stock Center, University of Indiana (BDSC)
AKH$^{-/-}$	n/a	S. Kondo, Tokyo University of Science
AKH-GAL4	25684	BDSC
AstC::2A::GAL4	845958	BDSC
CaLexA (*LexAop-CD8::GFP::2A::CD8::GFP; UAS-LexA::VP16::NFAT, LexAop-CD2::GFP/TM6B, Tb*)	66542	BDSC
Cg-GAL4	7011	BDSC
da-GAL4	55850	BDSC
Df(3L)delta130	n/a	A. von Philipsborn, Aarhus University
elav-GAL4	458	BDSC
NPF::2A::GAL4	84671	BDSC
NPFR::T2A::GAL4	n/a	S. Kondo, Tokyo University of Science
R57C10-GAL80-6 (on X)	n/a	R. Niwa, University of Tsukuba
SP^{0}	77892	BDSC
Tub-GAL80^{TS}	7108	BDSC
UAS-AKH-RNAi	105063	Vienna *Drosophila* Resource Center (VDRC)
UAS-LexA::VP16::NFAT; LexAop-Luciferase	n/a	M. Rosbash, Brandeis University
UAS-mCD8::GFP	5137	BDSC
UAS-Mondo-RNAi	109821	VDRC
UAS-NPF-RNAi^{KK}	108772	VDRC
UAS-NPF-RNAi^{sh}	330277	VDRC
UAS-NPF-RNAi^{TRiP}	27237	BDSC
UAS-NPFR-RNAi^{GD}	9605	VDRC
UAS-NPFR-RNAi^{TRiP}	25939	BDSC
UAS-SPR-RNAi	106804	VDRC
UAS-sut1-RNAi	65964	BDSC
UAS-sut2-RNAi	102028	VDRC
UAS-TrpA1	26263	BDSC
UAS-TrpA1[attP2]	n/a	C. Wegener, University of Würzburg
voilà-GAL4	n/a	A. Scopelliti, University of Glasgow
w^{1118}	60000	VDRC

Supplementary Table 2: Oligo sequences used for qPCR

**Primer name**
Rp49-F: AGTATCTGATGCCCAACATCG
Rp49-R: CAATCTCCTTGCGCTTCTTG
sut2-F*:* AGCATCGTTCCATCGCCATG
sut2-R*:* CATCAGAAGCGGGGICCATC
NPF-F: TGCAGTGTTGACATCGTTCTTTCG
NPF-R: AAACCGAAAGAACGATGTCAACAC
AstC-F: TACGGCCTACTCCTCACCC
AstC-R: GCTGGCATATTCGTAGCCACC
