# Supplementary material for: A gut-derived hormone suppresses sugar appetite and regulates food choice in Drosophila
Source: Nat Metab. 2022 Nov 7;4(11):1532–50. doi: 10.1038/s42255-022-00672-z (PMC9684077; doi:10.1038/s42255-022-00672-z)
Supplement: Supplementary file 2 — Reporting Summary [file 42255_2022_672_MOESM2_ESM.pdf]

nature portfolio

Corresponding author(s): Rewitz, K.

Last updated by author(s): September 22, 2022

Reporting Summary

Nature Portfolio wishes to improve the reproducibility of the work that we publish. This form provides structure for consistency and transparency in reporting. For further information on Nature Portfolio policies, see our Editorial Policies and the Editorial Policy Checklist.

Statistics

For all statistical analyses, confirm that the following items are present in the figure legend, table legend, main text, or Methods section.

n/a	Confirmed	
☐	☒	The exact sample size (*n*) for each experimental group/condition, given as a discrete number and unit of measurement
☐	☒	A statement on whether measurements were taken from distinct samples or whether the same sample was measured repeatedly
☐	☒	The statistical test(s) used AND whether they are one- or two-sided<br>*Only common tests should be described solely by name; describe more complex techniques in the Methods section.*
☒	☐	A description of all covariates tested
☐	☒	A description of any assumptions or corrections, such as tests of normality and adjustment for multiple comparisons
☐	☒	A full description of the statistical parameters including central tendency (e.g. means) or other basic estimates (e.g. regression coefficient) AND variation (e.g. standard deviation) or associated estimates of uncertainty (e.g. confidence intervals)
☐	☒	For null hypothesis testing, the test statistic (e.g. *F*, *t*, *r*) with confidence intervals, effect sizes, degrees of freedom and *P* value noted<br>*Give P values as exact values whenever suitable.*
☒	☐	For Bayesian analysis, information on the choice of priors and Markov chain Monte Carlo settings
☒	☐	For hierarchical and complex designs, identification of the appropriate level for tests and full reporting of outcomes
☒	☐	Estimates of effect sizes (e.g. Cohen's *d*, Pearson's *r*), indicating how they were calculated

Our web collection on statistics for biologists contains articles on many of the points above.

Software and code

Policy information about availability of computer code

Data collection	Zeiss Zen Blue v3.1 for image acquisition
Data analysis	FIJI/ImageJ vl.53<br>GraphPad Prism v9<br>Mathworks MATLAB 2021b, v9.11<br>Ctrax v0.5.18<br>FlyPAD v2.0 (http://www.flypad.pt/)<br>FLIC v1.0.0beta (https://github.com/PletcherLab/FLIC_R_Code).<br>The scripts used for image analysis are posted at https://doi.org/10.5281/zenodo.6641933 and https://doi.org/10.5281/zenodo.6641957.

For manuscripts utilizing custom algorithms or software that are central to the research but not yet described in published literature, software must be made available to editors and reviewers. We strongly encourage code deposition in a community repository (e.g. GitHub). See the Nature Portfolio guidelines for submitting code & software for further information.

Data

Policy information about availability of data

All manuscripts must include a data availability statement. This statement should provide the following information, where applicable:

- Accession codes, unique identifiers, or web links for publicly available datasets
- A description of any restrictions on data availability
- For clinical datasets or third party data, please ensure that the statement adheres to our policy

All data generated or analyzed during this study are available as Source Data files. Source data are provided with the paper.

Human research participants

Policy information about studies involving human research participants and Sex and Gender in Research.

Reporting on sex and gender	No human studies were performed.
Population characteristics	No human studies were performed.
Recruitment	No human studies were performed.
Ethics oversight	No human studies were performed. Ethical approval and oversight are not required for Drosophila studies.

Note that full information on the approval of the study protocol must also be provided in the manuscript.

Field-specific reporting

Please select the one below that is the best fit for your research. If you are not sure, read the appropriate sections before making your selection.

[x] Life sciences [ ] Behavioural & social sciences [ ] Ecological, evolutionary & environmental sciences

For a reference copy of the document with all sections, see nature.com/documents/nr-reporting-summary-flat.pdf

Life sciences study design

All studies must disclose on these points even when the disclosure is negative.

Sample size	Sample size was chosen based on similar previously published studies of Drosophila behavior and metabolism (doi.org:10.1038/s41467-022-28268-x, doi.org:10.1016/j.cmet.2018.09.021, doi.org:10.1038/s42255-020-0266-x, doi.org:10.1371/journal.pbio.2005004). No sample-size calculations were performed. The numbers of samples are large enough to capture normal variation while maintaining feasibility for preparation and are similar to or larger than those used in other published studies in the field. qPCR used 5-8 samples, each containing several tissues or animals, the standard in our lab; starvation and metabolic assays used approximately 10 replicates containing multiple animals. Image analyses made use of multiple tissues per genotype or condition, as described in the appropriate figure legends or methods.
Data exclusions	No data were excluded.
Replication	Representative images were chosen from multiple options, generally at least 6. All experiments producing numerical data include at least 5 replicates. All attempts at replication were successful.
Randomization	Animals were randomly grouped into batches as indicated in the text
Blinding	Researchers were not blinded during the study because this is not generally done in fly studies. With limited staff with expertise in these particular studies, the person handling sample prep must usually also be the one performing the assay.

Reporting for specific materials, systems and methods

We require information from authors about some types of materials, experimental systems and methods used in many studies. Here, indicate whether each material, system or method listed is relevant to your study. If you are not sure if a list item applies to your research, read the appropriate section before selecting a response.

Materials & experimental systems

n/a	Involved in the study	
☐	☒	Antibodies
☒	☐	Eukaryotic cell lines
☒	☐	Palaeontology and archaeology
☐	☒	Animals and other organisms
☒	☐	Clinical data
☒	☐	Dual use research of concern

Methods

n/a	Involved in the study	
☒	☐	ChIP-seq
☒	☐	Flow cytometry
☒	☐	MRI-based neuroimaging

Antibodies

Antibodies used

Rabbit anti-AKH, obtained from Jae Park (University of Tennessee), 1:500.
Rabbit anti-Allatostatin C (AstC), made by Jan Veenstra (University of Bordeaux), 1:500.
Mouse anti-GFP, ThermoFisher #A11120, 1:500.
Rat anti-mCherry, ThermoFisher #M11217, 1:2000.
Rabbit anti-NPF, RayBioTech #RB-19-0001-20, 1:500.
Mouse anti-Prospero, Developmental Studies Hybridoma Bank (University of Iowa) #MR1A, 1:20.
Alexa Fluor 488 goat anti-mouse, ThermoFisher #A32723, 1:500.
Alexa Fluor 555 goat anti-rabbit, ThermoFisher #A32732, 1:500.
Alexa Fluor 405 goat anti-rabbit, ThermoFisher #A31556, 1:500.
Alexa Fluor 555 goat anti-rat, ThermoFisher #A21434, 1:500.

Validation

Anti-AKH validated in Lee G and Park JH. (2004). Hemolymph sugar homeostasis and starvation-induced hyperactivity affected by genetic manipulations of the adipokinetic hormone-encoding gene in Drosophila melanogaster. Genetics 167, 311-323.

Anti-AstC validated in Veenstra JA, Agricola HJ, and Sellami A. (2008). Regulatory peptides in fruit fly midgut. Cell Tissue Res 334, 499-516.

Anti-NPF: valid for Drosophila according to sales page (https://www.raybiotech.com/rabbit-anti-npf-en/) and previous studies, e.g., "The Nutrient-Responsive Hormone CCHamide-2 Controls Growth by Regulating Insulin-like Peptides in the Brain of Drosophila melanogaster", Sano H et al., PLOS Genetics, May 28, 2015; "Developmental Ethanol Exposure Causes Reduced Feeding and Reveals a Critical Role for Neuropeptide F in Survival", Guevara A et al., Frontiers in Physiology, March 22, 2018.

Anti-GFP validated for staining by manufacturer (https://www.thermofisher.com/antibody/product/GFP-Antibody-clone-3E6-Monoclonal/A-11120).

Anti-mCherry validated for staining by manufacturer (https://www.thermofisher.com/antibody/product/mCherry-Antibody-clone-16D7-Monoclonal/M11217).

Anti-Prospero validated in "RK2, a glial-specific homeodomain protein required for embryonic nerve cord condensation and viability in Drosophila." Tomlinson A. Development (Cambridge, England) 120.10 (1994 Oct): 2957-66.

Goat anti-mouse, Alexa Fluor 488, validated by manufacturer (https://www.thermofisher.com/antibody/product/Goat-anti-Mouse-IgG-H-L-Highly-Cross-Adsorbed-Secondary-Antibody-Polyclonal/A32723).

Goat anti-rabbit, Alexa Fluor 555, validated by manufacturer (https://www.thermofisher.com/antibody/product/Goat-anti-Rabbit-IgG-H-L-Highly-Cross-Adsorbed-Secondary-Antibody-Polyclonal/A32732).

Goat anti-rabbit, Alexa Fluor 405, validated by manufacturer (https://www.thermofisher.com/antibody/product/Goat-anti-Rabbit-IgG-H-L-Cross-Adsorbed-Secondary-Antibody-Polyclonal/A-31556).

Goat anti-rat, Alexa Fluor 555, validated by manufacturer (https://www.thermofisher.com/antibody/product/Goat-anti-Rat-IgG-H-L-Cross-Adsorbed-Secondary-Antibody-Polyclonal/A-21434).

Animals and other research organisms

Policy information about studies involving animals; ARRIVE guidelines recommended for reporting animal research, and Sex and Gender in Research

Laboratory animals

This study made use of a variety of stocks of Drosophila melanogaster, detailed in the manuscript and Supplementary Table 2.
Most experiments examined female adults (5+ days after eclosion), with a few experiments including males as well.

Stocks created for this work:
10xUAS-IVS-myr::tdTomato[su(Hw)attP8]; CaLexA
AKH[ts]> (Tub-GAL80[TS]; AKH-GAL4)

AstC[gut]> (R57C10-GAL80; AstC::2A::GAL4)
EEC> (Tub-GAL80[TS]; voilà-GAL4)
NPF[gut]> (R57C10-GAL80; NPF::2A::GAL4)

Stocks obtained from Bloomington Drosophila Stock Center:
AKH-GAL4, #25684
AstC::2A::GAL4, #84595
CaLexA (LexAop-CD8::GFP::2A::CD8::GFP; UAS-LexA::VP16::NFAT, LexAop-CD2::GFP/TM6B, Tb), #66542
Cg-GAL4, #7011
da-GAL4, #55850
elav-GAL4, #458
NPF::2A::GAL4, #84671
SP[0], #77892
Tub-GAL80[TS], #7108
UAS-mCD8::GFP, #5137
UAS-NPF-RNAi[TRiP], #27237
UAS-NPFR-RNAi[TRiP], #25939
UAS-sut1-RNAi, #65964
UAS-TrpA1, #26263

Stocks obtained from Vienna Drosophila Resource Center:
UAS-AKH-RNAi, #105063
UAS-Mondo-RNAi, #109821
UAS-NPF-RNAi[KK], #108772
UAS-NPF-RNAi[sh], #330277
UAS-NPFR-RNAi[GD], #9605
UAS-SPR-RNAi, #106804
UAS-sut2-RNAi, #102028
w[1118], #60000

Others:
AKH[-] -- gift of S. Kondo, Tokyo University of Science.
Df(3L)delta130 -- gift of A. von Philipsborn, Aarhus University.
NPFR::T2A::GAL4 -- gift of S. Kondo, Tokyo University of Science.
R57C10-GAL80-6 (on X) -- gift of R. Niwa, University of Tsukuba.
UAS-TrpA1[attP2] -- gift of C. Wegener, University of Würzburg.
UAS-LexA::VP16::NFAT; LexAop-Luciferase -- gift of M. Rosbash, Brandeis University.
voilà-GAL4 -- gift of A. Scopelliti, University of Glasgow.

Wild animals	No wild animals were used in this study.
Reporting on sex	Our study identifies a hormonal pathway that appears to function differently in male and female Drosophila. Most experiments were performed with female animals, to study the female-specific function of the system. A few experiments were performed in males, which showed that the system functions differently in these animals, but no detailed followup was performed in males.
Field-collected samples	No field-collected animals were used in this study.
Ethics oversight	No ethics approval or oversight is required for Drosophila studies.

Note that full information on the approval of the study protocol must also be provided in the manuscript.
